# Abstracts from the International Symposium “Signal Transduction at the Blood–Brain Barriers” 2022

**DOI:** 10.1186/s12987-023-00417-4

**Published:** 2023-04-19

**Authors:** 

## A1 Extracellular vesicles-mediated intercellular communication at the blood–brain barrier

### Ibolya E. András, Hyung Joon Cho, and Michal Toborek

#### Department of Biochemistry and Molecular Biology, University of Miami Miller School of Medicine, Miami, FL 33,136, USA

##### **Correspondence:** Michal Toborek (mtoborek@med.miami.edu)

*Fluids and Barriers of the CNS* 2023, **20(Suppl 1)**: A1

Brain endothelial cells are potent producers of extracellular vesicles (EVs), and our research implicated endothelial-derived EVs in both HIV infection and amyloid pathology. We evaluated the proteome of EVs produced by brain endothelial cells and identified unique protein signatures of both the surface and the total EV proteome. Importantly, these signature proteins were modified by the exposure to HIV and/or elevated levels of amyloid. EV cargo can be efficiently transported by EVs from endothelial cells to the neighbouring cells of the neurovascular unit, including neural progenitor cells. Therefore, we analysed the impact of endothelial-derived EVs and EVs carrying Aβ (EV-Aβ) on gap junction communication between brain endothelial cells and neural progenitor cells (NPCs). Exposure to EV-Aβ resulted in significant reduction of connexin (Cx)43 and pCx43 protein expression in non-infected NPCs when compared to EV controls. Interestingly, EV-Aβ treatment significantly increased levels of Cx43, pCx43, and pannexin 2 in HIV-1-infected NPCs when compared to non-infected controls. These results were confirmed in a GJ functional assay and an ATP release assay, which is an indicator of connexin hemichannel and/or pannexin channel functions. Overall, our studies demonstrate the importance of endothelial-derived NPCs in the pathology of neurovascular unit, including aging and viral infection.

**Grant Support:** Supported by the Florida Department of Health Grant 8AZ24, the National Institutes of Health (NIH) Grants MH128022, MH122235, MH072567, HL126559, DA044579, DA039576, DA040537, DA050528, and DA047157.

## A2 Blood–brain barrier organoids and brain diseases—modelling cerebral malaria and Lyme neuroborreliosis induced barrier dysfunction

### Yvonne Adams^1^, Rebecca W. Olsen^1^, Peter Østrup Jensen^3^, Malin Lager^4^, Peter Wilhelmsson^5^, Anna J. Henningson^4,5,6^, Per-Eric Lindgren ^4,5^, Daniel Faurholt-Jepsen^7^, Helene Mens^7^, Kasper Nørskov Kragh^8,9^, Thomas Bjarnsholt^8,9^, Andreas Kjaer^2^, Anne-Mette Lebech^9,10^, and Anja R Jensen^1^

#### ^1^Centre for Medical Parasitology, Department of Immunology and Microbiology, Faculty of Health and Medical Sciences, University of Copenhagen, Copenhagen, Denmark; ^2^Department of Clinical Physiology and Nuclear Medicine & Cluster for Molecular Imaging, Copenhagen University Hospital-Rigshospitalet & Department of Biomedical Sciences, University of Copenhagen, Copenhagen, Denmark; ^3^Institute for Inflammation Research, Center for Rheumatology and Spine Diseases, Rigshospitalet, Copenhagen, Denmark; ^4^Division of Clinical Microbiology in Jönköping, Laboratory Medicine, Region Jönköping County, Department of Biomedical and Clinical Sciences, Linköping University, Linköping, Sweden;^5^Division of Inflammation and Infection, Department of Biomedical and Clinical Sciences, Linköping University, Linköping, Sweden; ^6^Division of Clinical Microbiology in Linköping, Department of Biomedical and Clinical Sciences, Linköping University, Linköping, Sweden; ^7^Department of Infectious Diseases, Copenhagen University Hospital, Rigshospitalet, Copenhagen, Denmark; ^8^Department of Clinical Microbiology, Rigshospitalet, Copenhagen, Denmark; ^9^Costerton Biofilm Center, Department of Immunology and Microbiology, Faculty of Health and Medical Sciences, University of Copenhagen, Copenhagen, Denmark; ^10^Department of Clinical Medicine, University of Copenhagen, Copenhagen, Denmark

##### **Correspondence:** Yvonne Adams (yadams@sund.ku.dk)

*Fluids and Barriers of the CNS* 2023, **20(Suppl 1)**: A2

The vector borne pathogens such as *Plasmodium falciparum* and *Borrelia burgdorferi *sensu lato can invade the central nervous system and cross the blood–brain barrier (BBB) leading to the most pathogenic forms of disease such as cerebral malaria (CM), and Lyme neuroborreliosis (LNB). CM is the most frequently fatal form of malaria where infection leads to BBB dysfunction and rapid onset swelling of the brain, resulting in compression of the brain stem [1, 2]. LNB infection triggers swelling of the meninges and painful meningoradiculitis [3,4]. Using 3D-cell culture we present a BBB-organoid model recapitulating the interactions between the components of the BBB (endothelial cells, pericytes and astrocytes). These cells self-assemble into organoids (250–350 µm in diameter) forming an “inside-out” model of the BBB, that mimics the in vivo environment. We challenged the organoids and measured the ability of the pathogens to bind, disrupt the BBB, and alter the gross morphology of the organoids using confocal microscopy. Challenging the BBB-organoids with the different pathogens resulted in alterations to barrier permeability, volume, and gross morphology in an isolate specific manner. This model represents a unique way to study the interactions between pathogens and the BBB, and to investigate the pathogenic effects of such events. These data represent for the first time a human-derived human model of the BBB which can be exploited to not only understand the molecular mechanisms driving migration across the BBB, but to develop better adjunctive treatments and chemotherapies to treat both CM and LNB.

## References


Seydel KB, Kampondeni SD, Valim C, Potchen MJ, Milner DA, Muwalo FW et al. Brain swelling and death in children with cerebral malaria. N Engl J Med. 2015;372(12):1126–37. https://doi.org/10.1056/NEJMoa1400116.Adams Y, Olsen RW, Bengtsson A, Dalgaard N, Zdioruk M, Satpathi S et al. Plasmodium falciparum erythrocyte membrane protein 1 variants induce cell swelling and disrupt the blood–brain barrier in cerebral malaria. J Exp Med. 2021;218(3):e20201266. https://doi.org/10.1084/jem.20201266.Hansen K, Lebech AM. The clinical and epidemiological profile of Lyme neuroborreliosis in Denmark 1985–1990. A prospective study of 187 patients with Borrelia burgdorferi specific intrathecal antibody production. Brain. 1992;(Pt 2):399–423. https://doi.org/10.1093/brain/115.2.399.Hansen K, Crone C, Kristoferitsch W. Lyme neuroborreliosis. Handb Clin Neurol. 2013;115:559–75. https://doi.org/10.1016/B978-0-444-52902-2.00032-1.

## A3 iPSC-derived neurovascular unit model to study neuroinvasion of parechovirus A3

### Pamela E. Capendale^1,2^, Inés García Rodríguez^1,2^, Lance Mulder^1,2^, Eline Freeze^1^, Renata Sá^1,3^, Adithya Sridhar^1,2^, Katja Wolthers^1*^,Dasja Pajkrt^1,2^*

#### ^1^OrganoVIR Labs, Amsterdam UMC location University of Amsterdam, Medical Microbiology, Amsterdam, the Netherlands; ^2^Amsterdam UMC location University of Amsterdam, Pediatric infectious Diseases, Amsterdam, the Netherlands; ^3^UniQure Biopharma BV, Amsterdam

##### **Correspondence:** Pamela Capendale (p.e.capendale@amsterdamumc.nl)

*equally contributed

*Fluids and Barriers of the CNS* 2023, **20(Suppl 1)**: A3

The blood brain barrier (BBB) protects the brain from various pathogens including viruses. However, some viruses do reach the central nervous system (CNS) and cause CNS diseases. Parechovirus A3 (PeV-A3) from the *Picornaviridae* family is among these CNS related viruses, however it is poorly understood how it crosses the BBB and infects the CNS. Here we develop a neurovascular unit (NVU) model comprised of iPSC-derived Blood Brain Barrier and Cerebral Organoids based on a Transwell system to study viral dynamics and the cellular tropism of PeV-A3, and the ability of the virus to enter the brain by infecting the BBB components. Brain Microvascular Endothelial Cells (BMEC), Pericytes, Astrocytes, Microglia and Cerebral Organoids (67 days) were generated separately from iPSC and inoculated with PeV-A3, PeV-A1 and Echo11. Production of viral particles and changes in cytokine expression were quantified using reverse transcriptase quantitative polymerase chain reaction (RT-qPCR).

Productive infection of cerebral organoids was observed by both PeV-A3, PeV-A1, and Echo11. However, upregulation of cytokine expression was only observed upon infection with PeV-A3 and Echo11, not PeV-A1. Results of infection of the BBB components show viral tropism of the PeV-A subtypes which demonstrates their ability to enter the CNS by infection of the BBB. Co-culture systems can influence the tropism of viruses and have previously enabled researchers to study human specific viruses on complex tissues such as the NVU. A representative human derived NVU model could give the long for desired insight in CNS related illness caused by PeV-A3.

## A4 From DNA methylation to protein function: selection and evaluation of regulated targets in human cerebral ischemia in vitro models of the blood–brain barrier

### Barbora Valentova, Sonja Peric, Lejla Hadzic, Iris Soleman, Lisa Frühbauer, Rebeka Halus, Anna Gerhartl, Lena Czeloth, Walter Pulverer, Karel Hanak, Klemens Vierlinger, Winfried Neuhaus

#### Competence Unit Molecular Diagnostics, AIT-Austrian Institute of Technology GmbH, Vienna, Austria

##### **Correspondence:** Winfried Neuhaus (winfried.neuhaus@ait.ac.at)

*Fluids and Barriers of the CNS* 2023, **20(Suppl 1)**: A4

Stroke and traumatic brain injury (TBI) are two threatening disorders of the central nervous system (CNS) that in case of survival usually result in serious permanent disabilities. Course of both diseases is accompanied by cerebral ischemia. Loss of blood–brain barrier (BBB) integrity is a consequence of cerebral ischemia. Recent studies demonstrated that the restorative processes initiated at the BBB after cerebral ischemia are regulated by epigenetic mechanisms.

Based on the investigation of changes in DNA methylation patterns of oxygen–glucose deprivation (OGD) treated cells of the neurovascular unit, new targets will be identified. The translation from DNA, via RNA, to the protein and functional levels should be investigated to define novel biomarkers and treatment targets. hCMEC/D3 cells, human primary astrocytes (hA) and pericytes (hP) were exposed to OGD for five hours and reoxygenated for further 19 h. Samples were analyzed for expression changes of seven DNA-methylation regulating enzymes (DNMTs, TETs, UHRF1) and subjected to EPIC arrays to analyze > 850.000 DNA-methylation sites per sample. Targets were chosen based on Kegg Pathway Database, Gene Set Enrichment Analysis, STRING database analysis and literature evaluation. Their expression was examined at mRNA and protein level by qPCR and western blotting, functional barrier relevance of targets was evaluated in OGD transwell models measuring transendothelial electrical resistance (TEER) and paracellular marker permeability. DNA-methylation regulating enzymes were significantly regulated due to OGD in hCMEC/D3, hA and hP at the mRNA as well as protein level. The effects were dependent on the cultivation set-up (mono-culture, co-cultures, triple-cultures). Based on DNA-methylation regulations, 40–50 targets were selected and the translation of their changes were confirmed at the mRNA level. From these, 13 targets were tested at protein level. Finally, addition of an inhibitor for a finally selected target blocked barrier breakdown during OGD. Data during this selection process were evaluated by analyzing selected targets also in samples from a second BBB model based on human induced pluripotent stem cell derived brain capillary endothelial-like cells and human TBI patient samples. It has been shown that DNA methylation analysis could be an excellent starting point for target identification in cerebral ischemia. Ultimately, translation of the DNA methylation signal to the mRNA and protein level enabled the identification and selection of a target whose blockade protected against BBB breakdown. In addition, the obtained data sets might emerge as valuable for understanding complex processes arising at the BBB during and after cerebral ischemia.

## A5 The effect of astrocyte-derived fatty acid-binding protein 7 on blood–brain barrier integrity in LPS-induced inflammation

### Deniz Altunsu^1,2^, Ecem Ayvaz^1,2^, Arzu Temizyürek^2^, Mehmet Kaya^2,3^, Bülent Ahıshalı^4^

#### ^1^Graduate School of Health Sciences, Koç University, Istanbul, Turkey; ^2^Research Center for Translational Medicine, Koç University Istanbul, Turkey; ^3^Department of Physiology and ^4^ Department of Histology and Embryology, School of Medicine, Koç University, Istanbul, Turkey

##### **Correspondence:** Deniz Altunsu (daltunsu19@ku.edu.tr)

*Fluids and Barriers of the CNS* 2023, **20(Suppl 1)**: A5

Sepsis is a life-threatening condition involving physiological, pathological, and biochemical abnormalities that can result from either exogenous factor derived by pathogens or endogenous factors released by the injured cells. Sepsis also affects the function of the various organs especially brain is more complicated than a systemic inflammatory response or immunological dysfunction. Brain capillary endothelial cells play a vital role in maintaining of the central nervous system homeostasis by regulating the passage of chemicals between blood circulation and brain parenchyma. Fatty acid-binding protein 7 (FABP7) regulates the production of caveolae, a specific type of lipid raft which functions in the transportation of molecules across the membrane in response to external stimuli by regulation of caveolin-1 protein. Moreover, FABP7 acts as a trophic factor and is involved in astrocytic differentiation and migration, thereby providing crucial support for vascular regeneration in the damaged brain. Although it regulates inflammatory signaling pathways, the role of FABP7 in lipopolysaccharide (LPS) induced inflammation has not been well studied yet. This study investigates the effect of exogenous FABP7 administration on LPS-induced blood–brain barrier (BBB) disruption in a sepsis mice model with a single dose of LPS (3 mg/kg). After an hour, LPS-injected mice were treated with FABP7 (40 and 80 μg/kg) for 24 h. Impact of the LPS and/or FABP7 on BBB permeability was assessed by injecting the fluorescent tracer Alexa Flour Albumin 594 after 23 h of LPS and/or FABP7 treatment. A single dose of LPS induced a significant tracer leakage into the brain tissue compared to the control mice administered with only saline. BBB leakage significantly decreased in 80 μg/kg administered with FABP7 compared to control and only LPS-treated mice (p < 0.05). Procalcitonin (PCT) levels in the LPS-injected mice significantly increased, where 80 µg/kg FABP7 importantly decreased (p < 0.05). A single dose of LPS increased the body temperature 24 h after the LPS administration, whereas a high dose of FABP7 decreased the body temperature from 41 °C to 37 °C. LPS severely triggered anxiety-like behavior in mice, where after treatment with a high dose of FABP7, anxiety-like behavior significantly decreased in LPS-induced mice model (p < 0.05). Our data demonstrated that time spent in the closed arm of elevated plus maze (EPM) is more extended than in the open arm/tendency time in LPS-induced mice compared to saline-treated mice. In conclusion, our data shows that FABP7 may be used as a potential therapeutic to treat sepsis-related dysfunctions.

## A6 Electronic cigarettes and alcohol cause mitochondrial stress via P2X7r in brain microvascular endothelial cells

### Naveen Mekala, Sachin Gajghate, Slava Rom, Uma Sriram, Nancy Reichenbach, Yuri Persidsky

#### Department of Pathology and Laboratory Medicine, Temple University Lewis Katz School of Medicine, Philadelphia, PA 19,140 USA

##### **Correspondence:** Yuri Persidsky (yuri.persidsky@tuhs.temple.edu)

*Fluids and Barriers of the CNS* 2023, **20(Suppl 1)**: A6

Electronic cigarettes (e‐Cig) use has increased steadily in recent years, especially among adolescents. Our prior studies showed that two months e‐cig inhalation led to cognitive demise, BBB leakiness accompanied by diminished expression of tight junction protein/glucose transporter, inflammatory responses in endothelium, and microglia activation [1]. The goal of this study was to investigate the injury mechanism of brain microvascular endothelial cells (BMVECs) by e‐Cig and ethanol (ETH) as both are used together**.** BMVECs were treated with ETH, acetaldehyde (ALD) or e‐Cig (0% or 1.8% nicotine) conditioned media. After 24 h, mitochondrial OXPHOS levels were measured by Seahorse machine (Agilent). Western blots (WB) were used to measure the expression of mitochondrial proteins. Gene expression (purinergic P2X_7_r and TRPV1) was investigated using qPCR. Intracellular Ca^2+^ was measured using calcium assay kit and ATP was detected by luminescent ATP Kit. There was 35% reduction in OXPHOS levels due to ETH, 45% by ALD and 50% by e‐Cig 1.8% nicotine (1.8% e‐Cig). Using qPCR, we observed a four‐fold increase in P2X7r expression after ETH, ALD and 1.8% e‐Cig. Expression of TRPV1 channels paralleled P2X7r showing a four‐fold increase after ETH or ALD, and six‐fold increase after1.8% e‐Cig exposure. BMVECs pre‐treated with P2X7r antagonist A804598 significantly reduced the expression of both P2X7r and TRPV1 channels. P2X7r antagonist preserved the mitochondrial function (OXPHOS) impaired by ETH, ALD and 1.8% e‐Cig, suggesting functional involvement of P2X7r in BMVEC injury caused by ETH or e‐Cig. WB revealed a 40% drop in Complex‐II (succinate dehydrogenase) expression and a 30% reduction in Complex‐IV (cytochrome *c* oxidase) expression after ETH, ALD or 1.8% e‐Cig exposure. We demonstrated significant increases in the intracellular Ca^2+^ levels after all insults except 0% e‐Cig. ETH and 1.8% e‐Cig exposure led to a thousand‐fold increase in intracellular Ca^2+^ levels, whereas ALD led to fifteen 100-fold the Ca^2+^ levels. ETH exposure caused a four‐fold increase in extracellular ATP release, while both ALD and 1.8% e‐Cig exposure showed eight‐fold increase in extracellular ATP concentrations. P2X7r antagonist significantly decreased Ca^2+^ release and ATP secretion as well as preserved expression of complex II and IV in mitochondria. Results indicate that ETH and e‐Cig share similar mechanism of BMVEC injury via mitochondrial impairment leading to intracellular Ca^2+^ and extracellular ATP releases. Further, we discovered a role of P2X7r inhibition in these harmful effects of ETH and e‐Cig suggesting novel therapeutic interventions.

## Reference


Heldt NA, Seliga A, Winfield M, Gajghate S, Reichenbach N, Yu X et al. Electronic cigarette exposure disrupts blood–brain barrier integrity and promotes neuroinflammation. Brain Behav Immun. 2020;88:363–380. https://doi.org/10.1016/j.bbi.2020.03.034.

## A7 A novel promising approach to target gut-brain axis and treat Alzheimer’s disease

### Carolina Pellegrini^1^, Rocchina Colucci^2^, Laura Benvenuti^1^, Vanessa D’Antongiovanni^1^, Clelia Di Salvo^1^, Simone Gastaldi^3^, Federica Blua^3^, Elisabetta Marini^3^, Barbara Rolando^3^, Loretta Lazzarato^3^, Antonio d’Amati^4,5^, Mariella Errede^5^, Daniela Virgintino^5^, Luca Antonioli^1^, Matteo Fornai^1^, Massimo Bertinaria^3^, Nunzia Bernardini^1^

#### ^1^Department of Clinical and Experimental Medicine, University of Pisa, Pisa, Italy; ^2^Department of Pharmaceutical and Pharmacological Sciences, University of Padova, Padova, Italy; ^3^Department of Drug Science and Technology, University of Turin, Italy; ^4^Department of Emergency and Organ Transplantation, Bari University School of Medicine, Bari, Italy; ^5^Department of Basic Medical Sciences, Neurosciences and Sensory Organs, Bari University School of Medicine, Bari, Italy

##### **Correspondence:** Carolina Pellegrini (carolina.pellegrini@unipi.it)

*Fluids and Barriers of the CNS* 2023, **20(Suppl 1)**: A7

Several evidences highlight the relevance of gut-brain axis in Alzheimer's disease (AD) [1]. In this context, the modulation of enteric inflammatory responses can represent a therapeutical approach to target gut-brain axis and treat AD [1]. We aimed to examine the effects of a gut-directed therapy in a spontaneous model of AD. Senescence-accelerated mouse prone 8 (SAMP8) mice (4 months old) were employed as an AD model and SAMR1 mice as controls. Mice were treated orally with a gut-directed NLRP3 inflammasome inhibitor (INF) 50 mg/kg/day for two months (n = 6/group) to evaluate the effects of a gut-directed therapy in early AD. Morris water maze test was performed to assess cognitive functions. Brain and colonic tissues were excised and processed for the evaluation of (1) AD protein levels [p-tau and Aβ1-42 by western blot (WB) and ELISA], (2) activation of inflammasome signaling (WB of caspase-1 and ELISA for IL-1β), (3) tight junctions (TJs) protein expression (WB). SAMP8 mice displayed cognitive dysfunctions, brain p-tau and Aβ1-42 accumulation, enteric inflammation and decreased colonic and brain TJ expression. INF treatment counteracted cognitive impairment and brain AD protein levels. INF also decreased enteric inflammasome signaling activation and prevented the decrease in TJ proteins expression in both gut and brain. The gut-directed NLRP3 inhibition exerts beneficial effects on early AD, counteracting cognitive dysfunctions, central AD-protein accumulation and preserving gut and brain barriers through the inhibition of enteric inflammation, suggesting the pharmacological modulation of NLRP3 in the gut as a promising strategy, targeting gut-brain axis, for AD treatment.

## Reference


Pellegrini C, Antonioli L, Calderone V, Colucci R, Fornai M, Blandizzi C. Microbiota-gut-brain axis in health and disease: Is NLRP3 inflammasome at the crossroads of microbiota-gut-brain communications? Prog Neurobiol. 2020;191:101806. https://doi.org/10.1016/j.pneurobio.2020.101806.

## A8 New insights on barrier function and discoveries of atypical NVU structures made with super-resolution microscopy applications

### Esti Sasson, Batia Bell, Shira Anzi, Aviv Halfon, Ayal Ben-Zvi

#### Department of Developmental Biology and Cancer Research, Faculty of Medicine, Hebrew University of Jerusalem, Jerusalem 91,120, Israel

##### **Correspondence:** Ayal Ben-Zvi (ayalb@ekmd.huji.ac.il)

*Fluids and Barriers of the CNS* 2023, **20(Suppl 1)**: A8

Stochastic optical reconstruction microscopy (STORM) for super-resolution imaging of barrier function through localization of proteins and tracer molecules in tissue samples, emerge as a robust tool for brain vasculature research. STORM combines high resolution together with the versatility of fluorescence microscopy, enabling Nano-scale molecular imaging of cellular components, and bridging classical use of electron microscopy and modern use of fluorescence microscopy. We developed STORM applications providing new insights on barrier function and discoveries of atypical neurovascular unit (NVU) structures. Here we present five discoveries made with this approach and focus on unpublished findings. First, developmental studies of blood–brain barrier (BBB) tight junctions (TJs) allow direct visualization of tracer leakage through immature TJs at very early stages of embryonic development, and shows that TJs’ restrictive properties to different substrates accrue at different developmental stages. Second, a new approach of 3D-STORM of isolated capillaries shed light on the dynamic changes in TJs ultrastructure along circadian cycles. Third, new degree of permeability across the arterial wall is deciphered using vesicular STORM imaging, to reveal atypical barrier function of endothelial and smooth muscle cells. Fourth, permeability of CVO vasculature to hormones is investigated with STORM imaging of fenestra. Finally, super-resolution imaging of basement membrane components take part in the discovery of a new neuro-vascular structure in the hippocampus. We believe that these findings are just the beginning of many more discoveries that will be made using STORM applications advancing our understanding of BBB function as well as exploring Nano-scale molecular changes during disease states.

## A9 Plasmin causes loss of barrier function and remodelling of key junctional molecules in brain microvascular monolayers

### James J. W. Hucklesby^1,2^, E. Scott Graham^2^, Catherine E Angel^1^

#### ^1^School of Biological Sciences, University of Auckland, Auckland, New Zealand; ^2^ Faculty of Medical Health Sciences, University of Auckland, Auckland, New Zealand

##### **Correspondence:** James Hucklesby (james.hucklesby@auckland.ac.nz)

*Fluids and Barriers of the CNS* 2023, **20(Suppl 1)**: A9

Tissue activated plasminogen (tPA) is used for the acute treatment of ischaemic stroke because it converts plasminogen to active plasmin that can break down clots. Previous studies show that tPA impairs endothelial barrier function through a variety of mechanisms, however, the direct effect of plasmin on the endothelial monolayer remains unclear. We aimed to determine the effect of plasmin on human cerebral microvascular endothelial cell (hCMEC/D3) monolayers. hCMEC/D3 monolayers cultured in serum-free media were treated with purified human plasmin. The endothelial barrier properties were monitored in real-time for 24 h using Electric Cell-substrate Impedance Sensing (ECIS). The expression and distribution of junctional molecules was assessed using immunocytochemistry; images were captured using an Operetta® CLS™ and analyzed using R. hCMVEC/D3 monolayers treated with plasmin exhibited a significant and concentration-dependent decline in barrier integrity, related to a reduction in cell-to-cell interactions. The inclusion of α2-antiplasmin significantly reduced this plasmin effect. Analysis of the immunocytochemistry images demonstrated that plasmin stimulated a redistribution or decline in key junctional molecules; plasmin stimulated a significant reduction in Claudin-5 and redistribution of ß-catenin away from the membrane and into the nucleus. Plasmin, a product of tPA treatment, impairs brain endothelial barrier function and therefore has important implications for tPA use in ischemic stroke patients.

## A10 Mutated in colon cancer protein (MCC): role in maintaining blood–brain barrier integrity through Wnt signaling

### Valentin Delobel, Béatrice Jaspard-Vinassa, Juliette Vaurs, Thierry Couffinhal, Cécile Duplàa

#### Biology of Cardiovascular Diseases, University of Bordeaux, Inserm, France

##### **Correspondence:** Valentin Delobel (valentin.delobel@inserm.fr)

*Fluids and Barriers of the CNS* 2023, **20(Suppl 1)**: A10

Blood brain barrier (BBB) disruption is critical for neurological disorders physiopathology. Wnt canonical signaling regulates BBB development and its stability. We have reported that excessive activation of the ubiquitin ligase PDZRN3, a Wnt canonical signaling inhibitor, destabilizes the BBB. However, the molecular pathway is still poorly understood. We aimed to identify novel Wnt signaling partners involved in BBB stabilization. Interacting effectors were searched by proximity labeling (BioID) in brain endothelial cells (EC). We employed lentiviral vector mediated overexpression and knockdown (KD) to manipulate gene expression in EC and performed biochemical studies to investigate signaling mechanism in vitro and in vivo. MCC, a tumor suppressor gene, was identified as a PDZRN3 interactant in EC. *Mcc* KD impairs directed EC migration in the flow direction and decreases EC permeability. In mature EC, MCC is mainly found in the phosphorylated state dependent of the Wnt-activated Caseine Kinase Iε (CKIε). We showed that PDZRN3 stabilizes MCC expression in EC, by blocking CKIε activity. During post-natal mouse BBB development, MCC undergoes a switch from un- to hyper-phosphorylated state. In mouse mutants, specific EC ectopic *Pdzrn3* expression reversed MCC phosphorylation correlated with BBB destabilization. This study discovered a Wnt-dependent post translation modification of MCC in EC, as a reversible process which dynamically regulates BBB stability.

## A11 Occludin regulates the antiviral RIG-1-like receptor signaling in human brain pericytes

### Silvia Torices, Nikolai Fattakhov, Kristyna Frydlova, Timea Teglas, Oandy Naranjo, and Michal Toborek

#### University of Miami Miller School of Medicine, Department of Biochemistry and Molecular Biology, Miami, FL, USA

##### **Correspondence:** Silvia Torices (storicesval@gmail.com)

*Fluids and Barriers of the CNS* 2023, **20(Suppl 1)**: A11

Occludin (ocln) is a tetraspan redox-sensitive protein associated with tight junctions of the blood–brain barrier (BBB) and it has been described as a multifunctional protein. By high-throughput RNA sequencing, we identified changes in gene expression-related to ocln modifications in human brain pericytes, one of the main regulatory cells of the BBB integrity. After ocln silencing, we found an alteration in several genes of the antiviral retinoic-acid-inducible gene-1 (RIG-1) signal pathway when compared with non-treated cells. RIG-1 is a cytosolic pattern recognition receptor (PRR) responsible for the interferon response after virus infection. Mechanistically, we provide evidence that cellular ocln level can modulate HIV-1 infection by controlling the expression levels of several interferon (INF)-stimulated genes such as ISG15, MX2, or IFIT1 through JAK/STAT signaling by influencing interferon regulatory factors (IRF) expression levels and STAT-1 activation. Furthermore, our results indicate that ocln can regulate mitochondrial dynamics and autophagy, potentially by its influence on the RIG-1 signaling pathway, which functions as a regulator of the cytoplasmic sensors of mitochondrial antiviral signaling protein (MAVS). Modulation of ocln expression levels can affect mitochondrial respiration and mitochondrial fission and fusion balance. Overall, these results are important to a better understanding of the molecular mechanisms for viral infection in the brain and describe previously unrecognized role of the protein ocln as a key factor in the control of innate immune response.

## A12 Protocadherin gamma C3 in breast cancer and melanoma and its role in interaction with brain microvascular endothelial cells

### Paul Glogau, Patrick Meybohm, Malgorzata Burek

#### Department of Anaesthesiology, Intensive Care, Emergency and Pain Medicine, University Hospital Würzburg, Würzburg, Germany

##### **Correspondence:** Malgorzata Burek (Burek_M@ukw.de)

*Fluids and Barriers of the CNS* 2023, **20(Suppl 1)**: A12

The blood–brain barrier (BBB) regulates brain homeostasis by providing a tight and selective barrier, but this is compromised in various diseases. We recently described Protocadherin gamma C3 (PCDHGC3) as one of the regulators of vascular function in brain microvascular endothelial cells (BMECs) [1, 2]. PCDHGC3 is expressed not only in BMECs but also in various cancer cells. Its role in the process of brain metastasis formation has not yet been characterized. We tested whether PCDHGC3 plays a role in the adhesion of melanoma and breast cancer cells to BMECs. These two types of cancers, along with lung cancer, are responsible for most brain metastases. We constructed PCDHGC3 knockout HCC1806 (a triple negative breast cancer cell line) and A2058 (melanoma cell line). Adhesion assays to hCMEC/D3 and CD34 + -derived brain like endothelial cells (BLECs) were performed using both knockout cell lines and their wild type counterparts. Changes in the expression of cell surface, extracellular matrix and metastasis markers were measured using real-time PCR. Wild type A2058 expressed high levels of PCDHGC3, while HCC1806 showed low PCDHGC3 expression. PCDHGC3 knockout resulted in a change in the growth and proliferation rate of both cell lines. PCDHGC3 knockout A2058 and HCC1806 adhered more strongly to hCMEC/D3 and BLECs. This correlated with an increased expression of matrix metalloproteinases-1 and -2 (MMP-1, -2). Our results suggest that PCDHGC3 is involved in the process of melanoma and breast cancer cell adhesion to BBB by affecting the expression of various genes involved in the process of cancer cell metastasis to the brain.

## References


Gabbert L, Dilling C, Meybohm P, Burek M. Deletion of Protocadherin Gamma C3 Induces Phenotypic and Functional Changes in Brain Microvascular Endothelial Cells In Vitro. Front Pharmacol. 2020;11:590144. https://doi.org/10.3389/fphar.2020.590144.Dilling C, Roewer N, Förster CY, Burek M. Multiple protocadherins are expressed in brain microvascular endothelial cells and might play a role in tight junction protein regulation. J Cereb Blood Flow Metab. 2017;37(10):3391–3400. https://doi.org/10.1177/0271678X16688706.

## A13 The differentiation degree of glioblastoma impacts on blood–brain barrier permeability

### Sabrina Digiovanni^1^, Iris Chiara Salaroglio^1^, Joanna Kopecka^1^, Pierre-Olivier Couraud^2^,Chiara Riganti^1^

#### ^1^Dipartimento di Oncologia, Università degli Studi di Torino, Torino; ^2^Institut Cochin, Université de Paris, Inserm U1016/CNRS, Paris, France

##### **Correspondence:** Sabrina Digiovanni (sabrina.digiovanni@unito.it)

*Fluids and Barriers of the CNS* 2023, **20(Suppl 1)**: A13

Glioblastoma multiforme (GBM) is a highly aggressive and chemoresistant tumor, difficult to treat with chemotherapy due to the blood–brain barrier (BBB), rich with tight junctions (TJs) and ATP-binding cassette transporters like P-glycoprotein (Pgp), which effluxes drugs out of the brain. BBB is disrupted in presence of GBM but the mechanisms are not known. We investigated whether the grade of GBM differentiation/stemness at the tumor-BBB interface influences the permeability of BBB. We co-cultured human brain endothelial capillary cells, hCMEC/D3, with 3 patient-derived GBM cells, as stem cells (SC) or differentiated cells (AC). The co-culture with GBM cells, in particular AC, increased the permeability to doxorubicin and dextran-70, compared to BBB alone, decreasing the expression of Pgp and TJ proteins. A secretome analysis identified IL-6 as significantly higher in AC than in SC medium. Notably, AC-medium treated with anti-IL-6 neutralizing antibody reduced the BBB permeability to SC level, SC- medium enriched with IL-6 increased BBB permeability to AC levels. IL-6 effects on permeability, Pgp and TJ expression is apparently mediated by STAT3 signaling in BBB cells. We suggest that the degree of GBM differentiation affects the production of IL-6, a BBB permeability modulator and a possible therapeutic target.

## A14 Impact of HER2 + brain-tropic breast cancer cells in blood–brain barrier permeability

### Liliana Santos^1^, Francesca Tomatis^2,3^, Hugo RS Ferreira^4,5^, José Sereno^1^, Sara F.F. Almeida^1^, Lino Ferreira^2,3^, Ana Paula Silva^4,5^, João Nuno Moreira^2,5^, Antero J. Abrunhosa^1^, Célia M. Gomes^4,5^

#### ^1^CIBIT/ICNAS-Institute for Nuclear Sciences Applied to Health, University of Coimbra, Portugal; ^2^CNC-Centre for Neuroscience and Cell Biology, University of Coimbra, Portugal; ^3^IIIUC-Institute of Interdisciplinary Research, University of Coimbra, Portugal; ^4^iCBR-Institute for Clinical and Biomedical Research, Faculty of Medicine, University of Coimbra, Portugal; ^5^CIBB-Center for Innovative Biomedicine and Biotechnology Consortium, University of Coimbra, Portugal

##### **Correspondence:** Liliana Santos (liliana.santos.ca26@gmail.com)

*Fluids and Barriers of the CNS* 2023, **20(Suppl 1)**: A14

Up to 50% of HER2^+^ breast cancer patients eventually develop brain metastasis (BM), with a median survival of less than 1 year after diagnosis. The formation of BM occurs through a complex multistep process, where breast cancer cells (BCCs) can modulate the brain microenvironment by secreting factors that will prepare the premetastatic niche (PMN) and favor their colonization. We aimed to evaluate the changes occurring in the BBB during the PMN formation. A BBB in vitro model was exposed to the secretome derived from HER2^+^ BCCs and their brain-tropic variants. BBB integrity was assessed by measuring the transendothelial flux of a 4 kDa-fluorescent dye, the TEER, and the expression of tight and adherens junction proteins. Nude mice were pretreated with the secretome obtained from brain-tropic cells and mouse models carrying BM or a primary orthotopic tumor were established. BBB integrity was assessed by near-infrared fluorescence imaging, and ex vivo by collagen IV and albumin immunostaining in the prefrontal cortex. The BBB was selectively disrupted in vitro and in vivo by brain-tropic cells indirectly through secreted factors. The presence of a localized primary tumor induced structural and functional alterations in the BBB integrity perceived by a decrease of collagen IV and an increase of albumin immunoreactivity, which allowed the passage of a 20 kDa dextran into the brain. Similar changes were observed in animals with BM. Our results demonstrate the active role of brain-tropic BCCs in the PMN formation, inducing dynamic changes in the BBB permeability for their subsequent colonization into the brain parenchyma.

## A15 Blood–Brain Barrier opening by Tumor Treating Fields (TTFields) is due to claudin-5 phosphorylation

### Ellaine Salvador^1^, Almuth F Kessler^1^, Malgorzata Burek^2^, Catherine Tempel Brami^3^, Tali Voloshin^3^, Alexandra Volodin^3^, Adel Zeidan^3^, Moshe Giladi^3^, Ralf-Ingo Ernestus^1^, Mario Löhr^1^, Carola Förster^2^, and Carsten Hagemann^1^

#### ^1^University Hospital Würzburg, Department of Neurosurgery, Section Experimental Neurosurgery, Josef-Schneider-Str. 11, D-97080 Würzburg, Germany; ^2^University Hospital Würzburg, Department of Anesthesiology, Section Experimental Anesthesiology and Medicine, Würzburg, Germany; ^3^Novocure Ltd., Haifa, Israel

##### **Correspondence:** Carsten Hagemann (Hagemann_C@ukw.de)

*Fluids and Barriers of the CNS* 2023, **20(Suppl 1)**: A15

We have recently reported that Tumor Treating Fields (TTFields), alternating electric fields approved for treatment of glioblastoma with a frequency of 200 kHz, can transiently open the blood–brain barrier (BBB) at 100 kHz frequency through claudin-5 delocalization [1]. Herein, we focus on understanding the mechanism through which this event takes place. Since TTFields are known to reorganize the microtubule network by activation of the guanine nucleotide exchange factor (GEF)-H1/Rho/ROCK signaling pathway, we investigated the involvement of this pathway in BBB opening via TTFields. Immortalized murine microvascular cerebellar endothelial cells (cerebEND) were treated with 100 kHz TTFields for 10–60 min, lysed for Western blot analysis and probed with antibodies against GEF-H1, as well as phosphorylated GEF-H1 and claudin-5. Similarly, the ROCK activity in the cells was measured using a ROCK activity assay kit. To further prove the effects of ROCK, the cells were treated with 10 μM of the ROCK inhibitor fasudil in combination with 100 kHz TTFields for 72 h, followed by claudin-5 staining for immunofluorescence microscopy. TTFields promoted a time-dependent increase in GEF-H1 phosphorylation with peaks after 10 min in cerebEND. Changes in claudin-5 phosphorylation were parallel to GEF-H1 activation. However, the elevated levels of claudin-5 phosphorylation remained relatively stable during the treatment period investigated. Subsequently, the ROCK activation in cerebEND was significantly increased after TTFields treatment. This is corroborated by inhibition of claudin-5 delocalization using fasudil. TTFields delocalize claudin-5 by phosphorylation brought about by GEF-H1 activation, leading to BBB opening.

## Reference


Voloshin T, Schneiderman RS, Volodin A, Shamir RR, Kaynan N, Zeevi E et al. Tumor Treating Fields (TTFields) Hinder Cancer Cell Motility through Regulation of Microtubule and Acting Dynamics. Cancers (Basel). 2020;12(10):3016. https://doi.org/10.3390/cancers12103016.

## A16 Improving brain delivery of the peptide-drug NA-1 through stabilization and conjugation to the “BBB homing” peptide BR1

### Hannah G. Kolberg, Amalie K. Andresen, Birger Brodin, Bente Gammelgaard, Mie Kristensen

#### Department of Pharmacy, University of Copenhagen, Denmark

##### **Correspondence:** Mie Kristensen (mie.kristensen@sund.ku.dk)

*Fluids and Barriers of the CNS* 2023, **20(Suppl 1)**: A16

Pharmacological treatment hindering brain tissue damage upon ischemic stroke is non-existant, though peptides that effectively hinder stroke-triggered neuronal death have been developed [1]. One example is NR2B9c, which is conjugated to the cell-penetrating peptide Tat to facilitate blood–brain barrier (BBB) permeation and neuronal uptake. However, Tat-NR2B9c (NA-1) does not enter the brain to an extent of clinical relevance [2]; possibly due to its poor plasma stability and broad biodistribution [3]. Here, we investigate whether NA-1 stabilization through synthesis with D-amino acids and NA-1 conjugation to the “BBB homing” peptide BR1 [4] will improve brain NA-1 delivery. L/D-NA-1 and L/D-BR1-NA-1 were labelled with selenomethionine to allow for sensitive detection using ICP-MS. Peptide stability was evaluated in mouse plasma and peptide brain uptake and biodistribution was assessed in mice.

D-NA-1 and D-BR1-NA-1 displayed better plasma stability when compared to their L-counterparts. The biodistribution study revealed that all NA-1-based constructs distributed broadly, thus questioning the “BBB homing” effect of BR1 when conjugated to NA-1. However, all the NA-1 constructs were detected in whole brain lysates, with L-BR1-NA-1 (0.52% ID/g) to a greater extent than L-NA-1 (0.25% ID/g), D-BR1-NA-1 (0.20% ID/g), and D-NA-1 (0.14% ID/g), when assessed via the selenium label. The D-NA-1-based constructs display better stability in plasma compared to their D-counterparts, whereas L-BR1-NA-1 displayed better brain uptake than its D-counterpart. Future pharmacodynamics studies will reveal whether inclusion of D-amino acids as well as BR1 conjugation will improve the therapeutic potential of the NA-1 constructs.

## References


Aarts M, Liu Y, Liu L, Besshoh S, Arundine M, Gurd JW et al. Treatment of ischemic brain damage by perturbing NMDA receptor- PSD-95 protein interactions. Science. 2002;298(5594):846–50. https://doi.org/10.1126/science.1072873.Hill MD, Goyal M, Menon BK, Nogueira RG, McTaggart RA, Demchuk AM et al. Efficacy and safety of nerinetide for the treatment of acute ischaemic stroke (ESCAPE-NA1): a multicentre, double-blind, randomised controlled trial. Lancet. 2020;395:878–887. https://doi.org/10.1016/S0140-6736(20)30258-0.Kristensen M, Kucharz K, Felipe Alves Fernandes E, Strømgaard K, Schallburg Nielsen M, Cederberg Helms HC et al. Conjugation of Therapeutic PSD-95 Inhibitors to the Cell-Penetrating Peptide Tat Affects Blood–Brain Barrier Adherence, Uptake, and Permeation. Pharmaceutics. 2020;12(7):661. https://doi.org/10.3390/pharmaceutics12070661.Körbelin J, Dogbevia G, Michelfelder S, Ridder DA, Hunger A, Wenzel J et al. A brain microvasculature endothelial cell-specific viral vector with the potential to treat neurovascular and neurological diseases. EMBO Mol Med. 2016;8(6):609–25. https://doi.org/10.15252/emmm.201506078.

## A17 Targets of microRNA-212/132 and their role at the blood–brain barrier

### Aili Sun, Laura Härtel, Patrick Meybohm, Malgorzata Burek

#### Department of Anaesthesiology, Intensive Care, Emergency and Pain Medicine, University Hospital Würzburg, Würzburg, Germany

##### **Correspondence:** Malgorzata Burek (Burek_M@ukw.de)

*Fluids and Barriers of the CNS* 2023, **20(Suppl 1)**: A17

Stroke is one of the leading causes of mortality and disability worldwide. MicroRNAs (miRs), short, non-coding, single-stranded RNA molecules, have been shown to play a role in stroke pathology and could be a promising therapeutic option in stroke. We have previously shown that miR-212/132 are elevated in hypoxic brain microvascular endothelial cells (BMECs) and suppress expression of their direct target genes claudin-1, junctional adhesion molecule 3 (JAM3) and tight-junction associated protein 1 (Tjap1) [1]. Here we further analyze the molecular mechanisms of action of miR-212/132 at the blood–brain barrier by knocking down their direct targets in BMECs. We stably transfected plasmids expressing shRNA targeting claudin-1, JAM3 or Tjap1 into BMEC cell lines cEND or cerebEND. After verifying suppression of gene and protein expression, we subjected the cell lines to Western blotting, real-time PCR and functional cellular assays. The BMEC cell line with suppressed Tjap1 expression showed lower mRNA expression of occludin, chemokine (C–C motif) ligand 2 (Ccl2), Ccl5, Ccl7, Colony stimulating factor 3 (Csf3), Claudin-10, Vascular cell adhesion molecule 1 (Vcam1), while Claudin-5, -12, Jam2, Tissue inhibitor of metalloproteinase 3 (Timp3) and VE-cadherin were elevated. No differences in expression of Tight junction protein 1 (Tjp1), ATP binding cassette subfamily B member 1 (Abcb1a), ATP binding cassette subfamily C member 1 (Abcc1) were observed. Interestingly, knocking down Tjap1 downregulated other miR-212/132 targets, claudin-1 and JAM3. Tjap1 knock down partially mimics the effects of elevated miR-212/132 on BBB. Tjap1 could contribute to stroke-induced changes observed at the blood–brain barrier, which will be investigated in further studies.

## Reference


Burek M, König A, Lang M, Fiedler J, Oerter S, Roewer N et al. Hypoxia-Induced MicroRNA-212/132 Alter Blood–Brain Barrier Integrity Through Inhibition of Tight Junction-Associated Proteins in Human and Mouse Brain Microvascular Endothelial Cells. Transl Stroke Res. 2019;10(6):672–683. https://doi.org/10.1007/s12975-018-0683-2.

## A18 Role of microRNA-223-3p overexpression in brain microvascular endothelial cells

### Marta Nowacka-Chmielewska^1^, Linus Homann^2^, Andrzej Malecki^1^, Patrick Meybohm^2^, Malgorzata Burek^2^

#### ^1^Laboratory of Molecular Biology, Institute of Physiotherapy and Health Sciences, Academy of Physical Education, Katowice, Poland; ^2^Department of Anaesthesiology, Intensive Care, Emergency and Pain Medicine, University Hospital Würzburg, Würzburg, Germany

##### **Correspondence:** Marta Nowacka-Chmielewska (m.nowacka@awf.katowice.pl), Malgorzata Burek (Burek_M@ukw.de)

*Fluids and Barriers of the CNS* 2023, **20(Suppl 1)**: A18

We and others identified that microRNA-223-3p was elevated in serum exosomes from people who underwent intensive physical exercise. MiR-223-3p has been shown to reduce the inflammatory response, but its role at the blood–brain barrier (BBB) has not yet been characterized yet. We examined the effects of overexpression of miR-223-3p in the human brain microvascular cell line hCMEC/D3 on BBB properties under normal and oxygen–glucose deprivation (OGD) conditions. The miR-223-3p expression plasmid containing Green Fluorescence Protein (GFP) was stably transfected into hCMEC/D3 cells. Transfected cells were selected with puromycin (2–4 µg/ml) for four weeks. MiR-223-3p overexpression was confirmed by qPCR/ GFP fluorescence. Cells were subjected to 4 h of OGD followed by a 24 h reoxygenation period. Expression analysis of BBB-related genes and proteins was performed using Western blot and qPCR. Paracellular permeability and transendothelial electrical resistance (TEER) were measured in control and miR-223-3p overexpressing cells. A high overexpression of miR-223-3p was detected in transfected hCMEC/D3 cells by qPCR. No significant differences in paracellular permeability and TEER could be detected in miR-223-3p overexpressing hCMEC/D3 compared to control. Claudin-5, HIF1a and VEGF mRNAs were significantly increased, while IL6 mRNA was decreased in miR-223-3p overexpressing hCMEC/D3 subjected to OGD compared to control. Our results suggest that miR-223-3p induced by physical activity or overexpressed in hCMEC/D3 can enhance the OGD-induced response by inhibiting inflammation and promoting angiogenesis, which could result in less secondary brain injury.

## A19 The role of pericytes in the asymmetrical effect of adenosine treatment on the blood–brain barrier paracellular tightness

### Lilla Barna^1,2^, Gabriela Hurtado-Alvarado^3^,András Harazin^1^,Krisztián Laczi^4^,András Kincses^1^,Gábor Rákhely^1,4^, Beatriz Gomez-Gonzalez^3^,Mária A. Deli^1^

#### ^1^Institute of Biophysics, Biological Research Centre, Szeged, Hungary;^2^Doctoral School in Biology, University of Szeged, Szeged, Hungary;^3^Department of Reproductive Biology, Metropolitan Autonomous University, Mexico City, Mexico;^4^Department of Biotechnology, University of Szeged, Szeged, Hungary

##### **Correspondence:** Lilla Barna (barna.lilla@brc.hu)

*Fluids and Barriers of the CNS* 2023, **20(Suppl 1)**: A19

Adenosine is an important signalling molecule that can regulate blood–brain barrier (BBB) tightness in pathological conditions. During sleep loss adenosine is accumulated, which via A2 adenosine receptor enhances BBB permeability and causes neuronal dysfunctions in rats. Our aim was to investigate the presence of adenosine receptors in the cells of the BBB and test the direct effect of adenosine on the BBB. We examined the expression of adenosine receptors by QRT-PCR. Cell viability was measured by impedance. BBB models were prepared from primary rat brain endothelial cells, glial cells and brain pericytes and were treated with adenosine and A2A receptor antagonist SCH-58261. The barrier function was tested by electrical resistance and permeability measurements on cultures and in rats in vivo. Rat brain endothelial cells expressed A2A, A2B receptors, rat pericytes expressed A1, A2A, A2B receptors, while astroglial cells expressed all four types. We demonstrated significant increase in impedance in adenosine treated groups in the first 2 h. Luminal adenosine treatment decreased the permeability in monoculture and co-culture BBB models, and decreased BBB permeability in rats treated by intracardiac adenosine injection. Abluminal adenosine treatment increased the permeability in the triple co-culture model indicating a role for pericytes. Adenosine given to the CSF increased the BBB penetration. SCH-58261 inhibited the effects of adenosine. We demonstrated the presence of adenosine receptors on all cell types of the BBB. Acute adenosine treatment from the luminal side tightened the barrier in BBB culture models and in rats. Brain pericytes may mediate the barrier opening effect of acute adenosine treatment from the abluminal side.

## A20 Brain-side transferrin modulates iron uptake at the blood–brain barrier

### Stephanie L. Baringer^1^, Elizabeth B. Neely^1^,Kondaiah Palsa^1^, Ian A. Simpson^2^, James R. Connor^1^

#### ^1^Department of Neurosurgery and ^2^Department of Neural and Behavioral Sciences, Penn State College of Medicine, Hershey, PA, USA

##### **Correspondence:** James R. Connor (jconnor@pennstatehealth.psu.edu), Stephanie L Baringer (slb820@psu.edu)

*Fluids and Barriers of the CNS* 2023, **20(Suppl 1)**: A20

Iron is crucial to the highly metabolically active brain. Fluctuations in brain iron levels can have detrimental effects on brain functioning, thus regulation of iron uptake into the brain at the level of the blood–brain barrier (BBB) is required. Here, using an in vivo steady state ventricular infusion, we demonstrate that increasing brain-side apo- (iron poor) transferrin (Tf) significantly increases ^55^Fe into the brain and microvasculature (p < 0.05). Additionally, we note that females do not have the same immediate ^55^Fe uptake response as males do. To investigate the mechanism of Tf’s regulation on iron release from endothelial cells (ECs) of the BBB, we employed hiPSC-derived ECs. ECs cultured on bi-chamber plates were exposed to apo- or holo (iron rich)-Tf in the basal (brain side) chamber. After cells were collected for western blotting, we found that holo-Tf reduced ferroportin (Fpn) levels by 50% (p < 0.05) and this decrease was prevented when inhibiting Fpn’s degradation. We are further investigating the direct interactions at play. Collectively, these results demonstrate that apo- and holo-Tf modulate iron release from ECs through interactions with Fpn. These data establish the mechanism for regulation of iron uptake into the brain and demonstrate sex-specific response to regulatory signals with implications for regional control.

## A21 Extracellular vesicles are involved in iron transport from human blood–brain barrier endothelial cells and are modified by iron status

### Kondaiah Palsa^1^, Stephanie Baringer^1^, Ganesh Shenoy^1^, Ian A. Simpson^2^, James R. Connor^1^

#### ^1^Department of Neurosurgery and^2^Department of Neural and Behavioral Sciences, Penn State College of Medicine, Hershey, Pennsylvania, USA

##### **Correspondence:** James R Connor (jconnor@pennstatehealth.psu.edu)

*Fluids and Barriers of the CNS* 2023, **20(Suppl 1)**: A21

We previously demonstrated that ferritin heavy chain (Fth1) transports iron to the brain via endothelial cells, identifying an alternate iron transport mechanism to the traditional transferrin system. Herein we investigated the role of extracellular vesicles (EV) in mediating the Fth1 or Transferrin (Tf) mediated iron transport across the blood–brain barrier (BBB) endothelial cells (ECs). The study used ECs derived from hiPSC that are grown in bicameral chambers. When cells were exposed to Tf-Fe^55^ or Fth1-Fe^55^, the Fe^55^ activity in the EV fraction in the basal chamber was significantly higher, compared to the supernatant fraction. Furthermore, the release of Tf, Fth1 and EVs membrane proteins (CD63, CD81) and EVs number is regulated by the iron concentration of the endothelial cells. Moreover, the EVs content of Tf and Fth1 was significantly higher when ECs were iron loaded compared to when they were iron deficient. The release of EVs containing iron bound to Tf or Fth1 was independent of hepcidin regulation indicating this mechanism by-passes a supposed major iron regulatory pathway. We further demonstrated that the EVs, containing Fth1 was taken up by co-cultured human astrocytes identifying a new pathway for these cells to obtain iron. These results indicate that iron transport across the BBB is mediated via the EVs pathway and is modified by iron status of the ECs providing evidence for novel alternate mechanisms of iron transport into the brain.

## A22 HIV infected pericytes and their influence on the extracellular signaling of the neurovascular unit

### Oandy Naranjo, Silvia Torices, Nikolai Fattakhov, Paul Clifford, Destiny Tiburcio, and Michal Toborek

#### Biochemistry and Molecular Biology, University of Miami Miller School of medicine, Miami, FL, USA

##### **Correspondence:** Oandy Naranjo (oxn62@miami.edu)

*Fluids and Barriers of the CNS* 2023, **20(Suppl 1)**: A22

The CNS was thought to be protected from HIV infection. However, experiments on microglia and astrocytes indicated that these cells are all capable of active and latent viral infection. Even on antiretroviral therapy (ART), HIV-infected individuals are at a higher risk for non-AIDS related co-morbidities, including neurological disease and stroke. BBB pericytes have been shown to regulate brain paracellular and transendothelial fluid transport at the BBB, maintain homeostasis of the CNS microenvironment, and maintain BBB integrity. Additionally, these cells possess the receptor profile enabling active HIV-1 infection. Due to their position between the periphery and the CNS we hypothesize that BBB pericytes are a key cell type for understanding the neuropathologies experienced by patients with HIV. We use a novel HIV reporter, named HIVGKO, that allows for purification of active HIV infected pericytes. Through fluorescent cell sorting and transcriptome analysis we were able to observe several dysfunctional pathways of actively infected pericytes and their noninfected counterparts within the same culture. Our transcriptome data along with functional assays will help in understanding how actively infected cells affect the delicate communication of the NVU.

**Grant Support:** The National Institutes of Health (NIH) grants MH128022, MH122235, MH072567, HL126559, DA044579, DA039576, DA040537, DA050528, and DA047157.

## A23 Method optimisation for in vitro small extracellular vesicles studies at the blood–brain barrier

### Adrián Klepe^*^, Ana Špilak^*^, Andreas Brachner, Christa Nöhammer, Winfried Neuhaus

#### Center for Health and Bioresources, Competence Unit Molecular Diagnostics, AIT Austrian Institute of Technology GmbH, Vienna, Austria

##### **Correspondence:** Winfried Neuhaus (winfried.neuhaus@ait.ac.at)

*equally contributed

*Fluids and Barriers of the CNS* 2023, **20(Suppl 1)**: A23

Small extracellular vesicles (sEVs) have a pivotal role in cell-to-cell communication, they are potential biomarkers and omnipresent in different body fluids. However, establishing methods for proper discrimination between sEVs and other particles is challenging [1]. Improvement of methods is highly needed to optimise the steps of sEV workflow for in vitro studies regarding the blood–brain barrier (BBB): from sEV isolation, quality, labelling, characterisation, to permeability and cell-EV interaction studies using e.g., transwell inserts. After sEV isolation (size exclusion chromatography, ultrafiltration) under adequate storage conditions (− 80 °C), discrimination between bona fide sEVs and other particles is essential [2]. Small EVs were fluorescently labelled (cell transfection, lipid-tracer dyes, membrane permeable dyes), simultaneously characterised by Western blotting for sEV markers; light scattering (NTA, flow cytometry) for concentration, size distribution, zeta potential; and sensitive microscopy-based methods for single particle analysis (correlative fluorescence cryo-electron microscopy, EVQuant®) [2]. Two BBB models were used: hCMEC/D3 immortalised cell line [3] and human induced pluripotent stem cell-derived brain capillary endothelial-like cells, hiPSC-BCECs [4]. EV labelling studies have revealed the importance of specific controls for single steps of EV preparation (controls of EV permeation, dye labelling, collection medium). Matrix coating volume and composition, properties of inserts (porosity, pore sizes) and the presence of serum are among the main influencing factors for permeation through both blanks and cell layers. Less than 1% of fluorescent EVs obtained with optimised protocols permeated across hCMEC/D3 and hiPSC-BCEC layers. Method development is indispensable to gain comparable results on EV permeation and EV interaction with cells in transwell BBB models. Controls are key elements since not all particles that scatter are EVs.

**Grant Support:** This work was founded by the European Union’s Horizon 2020 research and innovation programme (Marie Skłodowska-Curie project No 860303). We further gratefully acknowledge the financial support provided by the Austrian Science Fund FWF (project P 34137-B).

## References


Špilak A, Brachner A, Kegler U, Neuhaus W, Noehammer C. Implications and pitfalls for cancer diagnostics exploiting extracellular vesicles. Adv Drug Deliv Rev. 2021;175:113819. https://doi.org/10.1016/j.addr.2021.05.029.Théry C, Witwer KW, Aikawa E, Alcaraz MJ, Anderson JD, Andriantsitohaina R et al. Minimal information for studies of extracellular vesicles 2018 (MISEV2018): a position statement of the International Society for Extracellular Vesicles and update of the MISEV2014 guidelines. J Extracell Vesicles. 2018;7(1):1535750. https://doi.org/10.1080/20013078.2018.1535750.Gerhartl A, Pracser N, Vladetic A, Hendrikx S, Friedl HP, Neuhaus W. The pivotal role of micro-environmental cells in a human blood–brain barrier in vitro model of cerebral ischemia: functional and transcriptomic analysis. Fluids Barriers CNS. 2020;17(1):19. https://doi.org/10.1186/s12987-020-00179-3.Appelt-Menzel A, Cubukova A, Günther K, Edenhofer F, Piontek J, Krause G et al. Establishment of a human blood–brain barrier co-culture model mimicking the neurovascular unit using induced pluri- and multipotent stem cells. Stem Cell Reports. 2017;8(4):894–906. https://doi.org/10.1016/j.stemcr.2017.02.021.

## A24 Flow rectifier: a straightforward, low-cost system for studying brain microvascular endothelial cells (BMEC) under shear stress

### James J.W. Hucklesby^1,2^, Olivia Martin^2^, E. Scott Graham^2^, Catherine E. Angel^1^

#### ^1^School of Biological Sciences, University of Auckland, Auckland, New Zealand; ^2^Faculty of Medical Health Sciences, University of Auckland, Auckland, New Zealand

##### **Correspondence:** James Hucklesby (james.hucklesby@auckland.ac.nz)

*Fluids and Barriers of the CNS* 2023, **20(Suppl 1)**: A24

The importance of shear stress for maintaining a BMEC phenotype that is representative of BMECs in vivo has long been recognised. However, maintaining cells under shear stress in vitro remains highly technical. We aimed to design a low-cost straightforward system for culturing up to six hCMEC/D3 monolayers under physiologically relevant shear stress. Each Flow Rectifier loop consists of a syringe, tubing, 4 passive one-way valves and one channel of an Ibidi µSlide IV. When driven with a syringe pump, 3 ml of media is propelled around the circuit, providing continuous and unidirectional media flow. One syringe pump can drive multiple syringes, allowing 6 independent loops to simultaneously feed each of the slide’s 6 channels. hCMEC/D3s were cultured in an Ibidi µ-slide until confluent before exposure to shear stress for 12 h. Cellular morphology and phenotype were then assessed. The efficacy of ECIS flow array slides to study the barrier properties of hCMEC/D3s under shear stress was also explored. Following shear stress, hCMEC/D3s remained adherent, adopted a cobblestone-like morphology and remodelled key junctional molecules. The 6-channel setup enabled multiple treatments to be assessed simultaneously. The Flow Rectifier provides a more accessible solution for studying endothelial monolayers under shear stress.

## A25 A 3D iPSC-derived blood–brain barrier perfusable platform to vascularize organoids and investigate mechanisms of drug delivery to the brain

### Lena Jutz^1^, Colette Bichsel^1,2^, Martina Pigoni^1,2^, Gabrielle Py^1^, Iago Pereiro^2^, Julien Aubert^2^, Jose Luis Garcia Cordero^2^, Roberto Villaseñor^1^

#### ^1^Neuroscience and Rare Disease, Roche pRED, Basel, Switzerland; ^2^Roche Institute for Translational Bioengineering, Basel, Switzerland

##### **Correspondence:** Roberto Villaseñor (roberto.villasenor_solorio@roche.com)

*Fluids and Barriers of the CNS* 2023, **20(Suppl 1)**: A25

The application of brain organoids is limited by the lack of neurovascular unit interactions. Current organoid vascularization strategies still fail to recapitulate the organization found in vivo and reproducibility is a limitation. Additionally, the currently used endothelial cells show limited brain microvascular identity. Our goal is to create a microfluidic platform consisting of a patterned perfusable vessel in a hydrogel bed that can be combined with pericytes, astrocytes and eventually pre-vascularized brain organoids to achieve organoid perfusion. We designed a polydimethylsiloxane (PDMS) mold that includes an open-top hydrogel chamber where the vessel is formed and an inlet channel used to connect tubing for perfusion. In parallel, we developed a new protocol of iPSC endothelial cell (iEC) generation to optimize brain microvascular identity. Our plan is to seed iEC into the channels, together with gel-embedded pericytes and astrocytes, to achieve perfusable vessels in a brain microenvironment that can promote vascularization of brain organoids in the chip. The successful development of human blood brain barrier (BBB) models with improved brain endothelial cell identity will facilitate broader efforts for receptor/target discovery and will allow the modeling of pathological processes at the BBB.

## A26 Tumor treating fields (TTFields) reversibility disrupt the blood–brain barrier (BBB) in a human in vitro model

### Ellaine Salvador^1^, Almuth F. Kessler^1^, Theresa Köppl^1^, Sebastian Schönhärl^1^, Malgorzata Burek^2^, Ralf-Ingo Ernestus^1^, Mario Löhr^1^, Carola Förster^2^, Carsten Hagemann^1^

#### ^1^Section Experimental Neurosurgery, Department of Neurosurgery, University Hospital Würzburg, Würzburg, Germany; ^2^Department of Anesthesiology, University Hospital Würzburg, Würzburg, Germany

##### **Correspondence:** Carsten Hagemann (hagemann_c@ukw.de)

*Fluids and Barriers of the CNS* 2023, **20(Suppl 1)**: A26

The blood–brain barrier (BBB) impedes drug delivery to the CNS. Tumor Treating Fields (TTFields) are low intensity (1–3 V/cm), intermediate frequency (100–300 kHz) alternating electric fields, approved and effective for glioblastoma treatment at 200 kHz. Recently, we showed in vitro and in vivo in murine models that TTFields at lower frequencies transiently induce BBB permeability. We aimed to explore whether the transient opening of the BBB by TTFields translates to a human cell-based in vitro model. Primary human brain microvascular endothelial cells (HBMVEC) were cultured together with human pericytes. TTFields were applied for 24–96 h at 100–300 kHz, followed by a recovery period of 24–96 h. Transendothelial electrical resistance (TEER) was measured to analyze the effects on barrier integrity. Permeability of the barrier was assessed by quantifying the amount of FITC-dextran passing through the HBMVEC monolayer. Fractionated Western-blotting and immunofluorescence staining served to assess changes in expression and localization of the tight junction protein claudin-5, respectively. All investigated TTFields frequencies significantly decreased TEER across the HBMVEC monolayer. Effects started as early as 24 h and were strongest after 72 h at a TTFields frequency of 100 kHz. TTFields treatment delocalized claudin-5 from the cell boundaries to the cytoplasm. BBB recovery started as early as 24 h after TTFields cessation and was complete after 48 h. These results confirm our previous observations from murine models that TTFields could transiently open the BBB in a human cell culture model and demonstrate the feasibility of facilitating drug delivery to the CNS via concomitant application of TTFields.

## A27 A humanized neurovascular unit model to investigate leukocytes infiltration in multiple sclerosis

### Margherita Maria Ravanelli^1^, Elena Campo^2^, Giuseppe Liberatore^3^, Francesca Calcaterra^2,4^, Silvia Della Bella^2,4^, Domenico Mavillio^2,4^, Eduardo Nobile-Orazio^3,4^, Michela Matteoli^2,5^, Eliana Lauranzano^2^

#### ^1^Department of Biomedical Sciences, Humanitas University, Rozzano (Milan), Italy; ^2^Department of Biomedical Sciences, Humanitas Clinical and Research Center, Rozzano (Milan), Italy; ^3^Neuromuscular and Neuroimmunology Unit, IRCCS Humanitas Research Hospital, Rozzano (Milan), Italy; ^4^Department of Medical Biotechnologies and Translational Medicine, University of Milan, Milan, Italy; ^5^Institute of Neuroscience, CNR Institute of Neuroscience, Milan, Italy

##### **Correspondence:** Michela Matteoli (michela.matteoli@hunimed.eu)

*Fluids and Barriers of the CNS* 2023, **20(Suppl 1)**: A27

The blood–brain barrier (BBB) is a highly specialized barrier composed of cerebral endothelial cells (ECs) surrounded by pericytes and astrocytic endfeet forming the neurovascular unit (NVU). Progressive BBB breakdown and infiltration of autoreactive leukocytes into the CNS are central features in the pathogenesis of neuroinflammatory diseases such as Multiple Sclerosis (MS). We aim at developing up a humanized NVU in vitro model for addressing MS pathogenic molecular mechanisms and to characterize the phenotype of specific T cells subsets transmigrating the BBB in MS patients and healthy controls (HCs). Our NVU model consists of a contact co-culture of primary ECs and human astrocytes. Specifically, as a source of ECs, circulating endothelial colony forming cells (ECFCs) were isolated from the peripheral blood of MS patients and HCs. Autologous lymphocytes were used to perform patient-specific transmigration studies across the BBB model. We observed a significant increase in the in vitro appearance of ECFCs colonies isolated from naïve patients compared both controls and patients under pharmacological treatment. ECFCs’ phenotype was characterized confirming the expression of typical endothelial epitopes. Leukocytes transmigration across different NVU prototypes evidenced that human astrocytes presence during transmigration assay can influence the polarization of CD4 + T cells toward a Th1, Th1* and Th17 phenotype. Moreover, preliminary results highlighted a significantly greater transmigration of T cells isolated from MS patients compared to HCs. Our personalized NVU model demonstrated to be a new interesting tool to investigate the signature of T cells transmigrating the BBB in MS patients.

## A28 Human platelet lysate as a replacement for fetal bovine serum in a human blood–brain barrier in vitro model

### Andreas Brachner^1^, Christina Gruber^1^, Claudia Bernecker^2^, Peter Schlenke^2^, Winfried Neuhaus^1^

#### ^1^Center for Health and Bioresources, Competence Unit Molecular Diagnostics, AIT Austrian Institute of Technology GmbH, Vienna, Austria;^2^Department of Blood Group Serology and Transfusion Medicine, Medical University Graz, Graz, Austria

##### **Correspondence:** Winfried Neuhaus (winfried.neuhaus@ait.ac.at)

*Fluids and Barriers of the CNS* 2023, **20(Suppl 1)**: A28

Xenofree human cell culture is advantageous in view of scientific (batch variations, side effects caused by animal products) and ethical concerns, addressed by 3Rs (replacement, refinement, reduction of animal experimentation) societies, animal welfare organizations, and politics (directive 2010/63/EU). Aside animal experimentation per se, the replacement of animal products in in vitro experiments—particularly fetal bovine serum (FBS)—is an important goal. One alternative to FBS in cell culture is human platelet lysate (hPL), a by-product of transfusion medicine. In this study we evaluated the use of hPL in context of a human blood–brain barrier (BBB) model based on cell line hCMEC/D3. hPL was compared to FBS regarding support of hCMEC/D3 proliferation (short and long term cultivation) and barrier properties in a transwell model setup. Proliferation of cells cultivated in media containing FBS or hPL (O-in-AB) were compared in growth curves and cell cycle analyses (flow cytometry). Measured barrier properties of cells grown on transwell inserts included trans-endothelial electrical resistance (TEER) and carboxyfluorescein permeability. ABCB1 activity was determined by Calcein AM uptake upon inhibition of the transporter with Verapamil. Gene expression profiling was performed by Fluidigm high-throughput qPCR. Expression and localization of marker proteins were shown by immunofluorescence microscopy. Cultivation of hCMEC/D3 over 10 consecutive passages in hPL showed that proliferation is well supported by hPL, but at lower level as compared to FBS, which is also reflected in cell cycle distribution. The BBB model revealed that both, cells grown in hPL and FBS, reach a similar TEER value. The permeability coefficient for carboxyfluorescein was in both cases ca. 3 µm/min. Interestingly, the uptake of Calcein AM upon Verapamil treatment was higher in cells grown in hPL than in FBS, suggesting enhanced ABCB1 activity. Gene expression profiles were comparable, as well as expression and localization of marker proteins. Our study shows that hPL is an adequate alternative to replace FBS in hCMEC/D3 cell culture. Most of the evaluated BBB parameters are not changed in cells cultivated with hPL, in case of ABCB1 the transporter activity might be even enhanced by hPL.

## A29 Morphological and functional characterization of a human co-culture blood–brain barrier model and brain organoids in a microfluidic biochip

### Judit P. Vigh^1,2^, Anna Kocsis^1^, Ilona Gróf^1^, Lilla Barna^1^, Gergő Porkoláb^1,2^, Ana Raquel Santa-Maria^1^, András Kincses^1^, Sándor Valkai^1^, Silvia Bolognin^3^, Jens C. Schwamborn^3^, András Dér^1^, Mária A Deli^1^, Fruzsina R. Walter^1^

#### ^1^Institute of Biophysics, Biological Research Centre Szeged, EötvösLoránd Research Network, Szeged, Hungary; ^2^Doctoral School of Biology, University of Szeged, Szeged; ^3^Luxembourg Centre for Systems Biomedicine, University of Luxembourg, Belvaux, Luxembourg

##### **Correspondence:** Fruzsina Walter (walter.fruzsina@brc.hu), Maria Deli (deli.maria@brc.hu)

*Fluids and Barriers of the CNS* 2023, **20(Suppl 1)**: A29

Microfluidic chip devices allow the more complex and physiological modelling of the blood–brain barrier (BBB). In previous generations of BBB models mostly astrocytes or mixed glial cells represented the brain compartment. The iPSC technology resulted in the availability of human brain spheroids and organoids that provide a simplified 3D model of the central nervous system including different types of neurons. We aimed to (1) examine the interaction between a human BBB model and midbrain organoids using a static setup and (2) develop a microfluidic device to co-culture BBB cells and brain organoids for morphological and functional experiments. Human endothelial cells and bovine pericytes were co-cultured to establish the human BBB model. Brain organoids were differentiated from healthy (WT) and Parkinson disease patients’ (PD) iPS cells. To examine the barrier integrity, we performed transendothelial electrical resistance (TEER), permeability measurement and immunostaining for tight junction proteins in culture inserts. The presence of brain organoids (WT and PD, 2-day co-culture) did not influence the barrier integrity—TEER, permeability and claudin-5 immunostaining- of the human BBB model in static conditions. We successfully integrated the brain organoid compartments into the biochips and characterized both the BBB model and the brain organoids (βIII-tubulin, MAP-2, GFAP immunostainings). A BBB-brain organoid co-culture model with good barrier integrity was established and tested for nanoparticle permeability. We successfully developed a new microfluidic device and co-cultured a human BBB model and brain organoids. This complex organ-on-a-chip system can be a valuable tool for further experiments.

**Grant Support:** NTP-NFTÖ-22-B-0229 Hungarian fellowship, 141547 MEC_R Hungarian fellowship (JPV), National Research, Development and Innovation Office of Hungary (Grant no. NNE 129617—M-ERA.NET2 nanoPD; MAD), Eötvös Loránd Research Network (Grant no. SA-111/2021; FRW).

## A30 Investigation of monocyte migration due to neuroinflammation through an in vitro BBB model

### Zehra Gül Morçimen^1^, Barış Güliçli^1^, Aylin Şendemir^1,2^

#### ^1^Ege University, Graduate School of Natural and Applied Sciences, Department of Bioengineering, İzmir, Turkey; ^2^Ege University, Graduate School of Natural and Applied Sciences, Department of Biomedical, Technologies, İzmir, Turkey

##### **Correspondence:** Zehra Gül Morçimen (zehramorcimen@gmail.com)

*Fluids and Barriers of the CNS* 2023, **20(Suppl 1)**: A30

The blood–brain barrier (BBB) acts as a selective semipermeable barrier separating the central nervous system (CNS) from the circulatory system [1]. Circulating immune cells normally do not enter the healthy brain unless the BBB is damaged [2]. Despite the immune privilege, a physical deterioration may occur in the BBB due to reasons such as inflammation, hypertension, autoimmunity, and cancer, and cause circulatory immune cells to enter CNS and increase inflammation. We aimed to clarify the complex relationship of BBB and neuroinflammation through the simulation of monocyte migration through the BBB. In vitro BBB model was created using bacterial cellulose (BC) as basal membranes. BC pore size was modified using polycaprolactone microspheres. BCs were shown to have a pore diameter of 4–10 µm. Human brain microvascular endothelial cells were seeded in the luminal portion of the Cell Crown inserts, and tight junctions were followed by TEER measurements. Neuroinflammation model was created on thrice-subcloned cell line derived from the SK-N-SH neuroblastoma (SHSY-5Y) cells by hydrogen peroxide treatment in the abluminal part. After reaching the target TEER value and inflammation level, fluorescently labeled monocyte cells were introduced from the luminal part, and their migration to abluminal part was evaluated. Neuroinflammation effected TEER vales, BBB permeability and monocyte migration rate. Differences in TEER values and monocyte migration reveal the relationship between neuroinflammation and BBB.

**Grant Support:** This study was funded by the Scientific and Technological Research Council of Turkey (TUBITAK) ARDEB 1001 Grant No 221M092.

## References


Carson MJ, Doose JM, Melchior B, Schmid CD, Ploix CC. CNS immune privilege: hiding in plain sight. Immunol Rev. 2006;213:48-65. https://doi.org/10.1111/j.1600-065X.2006.00441.x.Yin J, Valin KL, Dixon ML, Leavenworth JW. The Role of Microglia and Macrophages in CNS Homeostasis, Autoimmunity, and Cancer. J Immunol Res. 2017;2017:5150678. https://doi.org/10.1155/2017/5150678.

## A31 A novel cellular in vitro model of Alzheimer’s disease blood–brain barrier

### Giulia Sierri^1^, Silvia Sesana^1^, Claudia G. Almeida^2^, Wiep Scheper^3^, Thomas G. Ohm^4^, Massimo Masserini^1^, Carlo Ferrarese^1^, Sandrine Bourdoulous^5^, Francesca Re^1^

#### ^1^School of Medicine and Surgery, University of Milano-Bicocca, Monza, Italy; ^2^Universidade NOVA de Lisboa, Lisboa, Portugal; ^3^VU University Medical Center, Amsterdam, The Netherlands; ^4^Department of Clinical Cell and Neurobiology, Institute of Integrative Neuroanatomy, CC2, CharitéUniversitätsmedizin Berlin, Berlin, Germany; ^5^Institut Cochin, Inserm U1016, CNRS UMR8104, Université Paris Descartes, Paris, France

##### **Correspondence:** Francesca Re (francesca.re1@unimib.it)

*Fluids and Barriers of the CNS* 2023, **20(Suppl 1)**: A31

A major challenge in diagnosis and therapy of neurodegenerative diseases is the crossing of the blood–brain barrier (BBB). In vitro BBB models are available as a screening platform [1], but they only mimic healthy conditions without taking into account the BBB alterations associated with neurodegenerative diseases [2]. The overexpression of the receptor for advanced glycation end-products (RAGE) transporting β-amyloid peptide (Aβ) into the brain from the blood, and of claudin-5, a tight junction protein sealing the brain capillary endothelial wall, and the down-regulation of low-density lipoprotein-related protein-1 (LRP1) receptor and of P-glycoprotein (Pgp), transporting Aβ from the brain to the blood have been described in brain of Alzheimer (AD) subjects [3]. A monolayer of human cerebral microvascular endothelial cells (hCMEC/D3) cultured on transwell inserts was used as model of a healthy BBB. Alterations of the properties of this barrier induced by AD fluids have been investigated upon incubation with (i) Aβ peptide, (ii) conditioned media from neuron-like SHSY5Y cells stably expressing APP with the Swedish mutation, (iii) conditioned media from neuron-like SK-N-SH cells expressing wild-type or FTD mutant tau, or (iv) CSF and plasma from AD patients. hCMEC/D3 cells stably overexpressing RAGE or downregulating LRP1 have been also developed. The results obtained demonstrated a major involvement of Aβ peptide in BBB modifications. Of most interest, the effects of Aβ on the structural and functional features of hCMEC/D3 cells were different if the peptide was present in the ‘blood’-side or in the ‘brain’-side. When Aβ was present in the brain side the tightness of the barrier increased, as shown by a decrease of the blood-to-brain permeability and the increase of TEER. Contrarily, the tightness decreased when Aβ was present in the blood side. When hCMEC/D3 cells were transfected to stably overexpressed RAGE, then cells were also found to spontaneously overexpress claudin-5, with the result of increasing the tightness of the barrier, decreasing the blood-to-brain permeability and increasing the TEER. Altogether, the results so far obtained suggest that, in AD, either the altered composition of biological fluids or the molecular changes present in endothelial cells, are inducing a reduced permeability of the BBB. The BBB models developed could help to accelerate the screening and discovery of novel drugs for treatment of neurodegenerative disorders.

**Grant Support:** This work was supported by Joint Programme-Neurodegenerative Disease Research (JPND Research 2015) to F Re (CUP B42F16000090008).

## References


Williams-Medina A, Deblock M, Janigro D. In vitro Models of the Blood–Brain Barrier: Tools in Translational Medicine. Front Med Technol. 2021;2:623950. https://doi.org/10.3389/fmedt.2020.623950.Magro RD, Cox A, Zambelli V, Mancini S, Masserini M, Re F. The ability of liposomes, tailored for blood–brain barrier targeting, to reach the brain is dramatically affected by the disease state. Nanomedicine (Lond). 2018;13(6):585–594. https://doi.org/10.2217/nnm-2017-0317.Yamazaki Y, Kanekiyo T. Blood–Brain Barrier Dysfunction and the Pathogenesis of Alzheimer's Disease. Int J Mol Sci. 2017;18(9):1965. https://doi.org/10.3390/ijms18091965.

## A32 Non-classical monocytes promote neurovascular repair in small vessel disease associated with microinfarctions

### Sarah Lecordier, Romain Menet, Anne-SophieAllain, Ayman ElAli

#### Research Centre of CHU de Québec, Université Laval, Quebec City, QUEBEC, Canada

##### **Correspondence:** Ayman ElAli (ayman.el-ali@crchudequebec.ulaval.ca)

*Fluids and Barriers of the CNS* 2023, **20(Suppl 1)**: A32

Cerebral small vessel disease (cSVD) is increasingly recognized as a major risk factor for dementia. cSVD comprises a varied group of cerebrovascular pathologies causing micro-lesions associated with neurovascular deregulation and neuroinflammation, leading to cognitive decline. Inflammatory cells, mainly monocytes and microglia, play important roles in modulating the injury and repair processes in brain injuries. Herein, we aimed to investigate the contribution of non-classical C-X3-C motif chemokine receptor (CX3CR)1^+^ monocytes to the pathobiology and therapy of cSVD. To this end, we generated chimeric mice in which bone marrow derived CX3CR1^+^ monocytes expressing green fluorescent protein (GFP) are either functional or dysfunctional. Mice were subjected to cSVD via occlusion of the penetrating cerebral arterioles. Furthermore, novel pharmacological approaches were used to modulate the function of CX3CR1^+^ monocytes. Our findings demonstrate that CX3CR1^+^ monocytes transiently infiltrated the lesioned hippocampus and were recruited to the microinfarcts 7 days after cSVD. The infiltration rate inversely correlated with neuronal degeneration and blood–brain barrier (BBB) disruption. Generation of dysfunctional CX3CR1^+^ monocytes exacerbated microinfarctions and cognitive decline, associated with impaired microvascular structure in the lesioned hippocampus. Pharmacological stimulation of CX3CR1^+^ monocyte generation attenuated neuronal loss and improved cognitive functions by promoting microvascular density as well as function translated by enhanced cerebral blood flow (CBF). These changes were associated with elevated levels of pro-angiogenic factors and matrix stabilizers in the blood circulation. The results indicate that CX3CR1^+^ monocytes promote neurovascular repair after cSVD, outlining the promises of targeting these cells for the development of new therapies.

## A33 Physical activity-based strategy to protect against methamphetamine-induced neurotoxicity

### Arkadiusz Liśkiewicz^1^, Marta Przybyła^1^, Minseon Park^2^, Andrzej Małecki^1^, and Michal Toborek^1,2^

#### ^1^Institute of Physiotherapy and Health Sciences, The Jerzy Kukuczka Academy of Physical Education, Katowice 40065, Poland; ^2^Department of Biochemistry and Molecular Biology, University of Miami Miller School of Medicine, Miami, FL 33136, USA

##### Correspondende: Andrzej Małecki (a.malecki@awf.katowice.pl), Michal Toborek (mtoborek@med.miami.edu)

*Fluids and Barriers of the CNS* 2023, **20(Suppl 1)**: A33

Among the pathomechanisms of substance use disorders, the disruption and increased permeability of the blood–brain barrier (BBB) has been recently recognized. Indeed, we identified endothelial dysfunction and disruption of the BBB as prominent events associated with long-term methamphetamine neurotoxicity and methamphetamine-mediated aberrant neurogenesis of neural progenitor cells via IL-1beta (interleukin-1 beta), the effector cytokine of the inflammasome activation. Physical exercise is a relatively inexpensive and feasible way to implement behavioral therapy counteracting the methamphetamine-induced BBB impairment. While physical activity impacts brain functions, the mechanisms of this effect are not fully recognized or understood. We performed global metabolomics profiling of the hippocampus and the frontal cortex (FC) in a model of voluntary running in mice. Examined brain structures responded differentially to physical activity. The voluntary wheel running differently modulated the hippocampal and cortical composition of metabolites ensuring higher cellular energetic demands. Increased physical activity was associated with upregulation of hippocampal lipogenesis and highly specific alterations in fatty acids (FA) composition. Simultaneously exercise resulted in less anxious behavior. Altogether, our study links physical activity to highly specific changes in brain metabolite profile that can affect behavioral modifications.

**Grant Support:** Supported by the National Science Centre (NSC) Grants 2015/17/B/NZ7/02985and 2019/35/B/NZ7/03155.

## A34 Investigation of mitochondrial function in cell models of *PARK2* copy number variation carriers with adult ADHD

### Markus Glaser^1^, Zora Schickardt^2^, Sabrina Oerter^1,3^, Rhiannon McNeill^2^, Carolin Koreny^2^, Sarah Kittel-Schneider^2^ and Antje Appelt-Menzel^1,3^

#### ^1^Chair Tissue Engineering and Regenerative Medicine, University Hospital Würzburg, Würzburg, Bavaria, Germany; ^2^Department of Psychiatry, Psychosomatics and Psychotherapy, University Hospital Würzburg, Würzburg, Bavaria, Germany; ^3^Translational Center Regenerative Therapies TLC-RT, Fraunhofer Institute for Silicate Research ISC, Würzburg, Bavaria, Germany

##### **Correspondence:** Markus Glaser (markus.glaser@uni-wuerzburg.de)

*Fluids and Barriers of the CNS* 2023, **20(Suppl 1)**: A34

Copy number variations (CNVs) in the *PARK2* gene are associated with attention deficit hyperactivity disorder (ADHD) [1]. Thus, genetic variants in *PARK2* might result in protein dysfunction, thereby impacting mitochondrial stability. Blood–brain barrier (BBB) disruption has been implicated in ADHD [2], since changes in BBB functionality and metabolic activity can lead to barrier opening and a disrupted microenvironment, impairing neuronal physiology. Taken together, there appears to be a link between ADHD, mitochondrial and BBB dysfunction.

The aim of the project is to establish an advanced isogenic in vitro test system of the neurovascular unit (NVU) combining the BBB with the adjacent central nervous system to recapitulate ADHD-related cellular and molecular changes. Furthermore, we aim to provide a standardized platform for drug screening in ADHD treatment. Fibroblasts of *PARK2* CNV carriers and healthy controls were reprogrammed in human induced pluripotent stem cell (hiPSC) lines. According to established differentiation protocols, hiPSC-derived brain capillary-like endothelial cells (BCECs) and cortical neurons were generated and characterized. Therefore, specific marker expression, barrier integrity, electrical potentials, mitochondrial biogenesis, morphology and energetics were assessed. Patient-specific BCECs and cortical neurons were co-cultured in static transwell-based setups and subsequently under microfluidic culture conditions. Compared to respective mono-culture, complex crosstalk between both cell types was examined. Additionally, shear stress-induced effects on the BBB phenotype could be investigated. The NVU model can be used to replicate and explore gene-environment interactions by simulating ADHD-associated risk factors. Furthermore, therapeutic approaches will be validated to restore mitochondrial and cellular function.

## References


Jarick I, Volckmar AL, Pütter C, Pechlivanis S, Nguyen TT, Dauvermann MR et al. Genome-wide analysis of rare copy number variations reveals PARK2 as a candidate gene for attention-deficit/hyperactivity disorder. Mol Psychiatry. 2014;19(1):115–21. https://doi.org/10.1038/mp.2012.161.Leffa DT, Torres ILS, Rohde LA. A Review on the Role of Inflammation in Attention-Deficit/Hyperactivity Disorder. Neuroimmunomodulation. 2018;25(5–6):328–333. https://doi.org/10.1159/000489635.

## A35 MSCs induce beneficial effects in EAE mice: how do they do it?

### Antonio d’Amati^1,4^, Mariella Errede^1^, Tiziana Annese^1^, Francesco Girolamo^1^, Ignazio de Trizio^1^, Antonio Uccelli^2,3^, Nicole Kerlero de Rosbo^3,5^, Daniela Virgintino^1^

#### ^1^Department of Basic Medical Sciences, Neuroscience, and Sensory Organs, University of Bari, Bari, Italy. ^2^Department of Neurosciences, Ophthalmology, Rehabilitation, Ophthalmology, Genetics, Maternal and Child Health (DINOGMI), University of Genoa, Genoa, Italy. ^3^IRCCS OspedalePoliclinico San Martino, Genova, Italy. ^4^Department of Emergency and Organ Transplantation, Pathology Unit, University of Bari, Bari, Italy. ^5^TomaLab, Institute of Nanotechnology, Consiglio Nazionale delle Ricerche (CNR), Rome, Italy

##### **Correspondence:** Antonio d’Amati (antonio.damati@uniba.it)

*Fluids and Barriers of the CNS* 2023, **20(Suppl 1)**: A35

The cerebral cortex of mice with experimental autoimmune encephalomyelitis (EAE) is characterized by neuroinflammation associated with blood–brain barrier (BBB) impairment. In this condition, the administration of mesenchymal stem cells (MSCs) induces evident beneficial effects through their anti-inflammatory and reparative activities that were recently associated with the release by MSCs of extracellular vesicles (EVs). EVs are nano-sized structures that are released into the blood, carrying various molecules (e.g., lipids, proteins, DNA, mRNA, miRNA), have similar functions as their cells of origin, and modulate immune responses in various diseases. However, little is known about their release in vivo and the possible role of MSC-derived EVs in EAE. In this study we have analyzed the localization in the lungs of MSCs injected in EAE-affected mice. A pulmonary first-pass role occurs upon intravenous administration of MSCs, when over 70% of MSCs accumulate in the lungs. In addition, physical interactions with the pulmonary tissue/capillaries and the possible release of EVs at this site have been assessed to study the potential correlation between MSC-derived EVs and the beneficial effects exerted by MSCs in EAE. Immunochemical analyses were carried out on cerebral cortex and lungs of EAE-affected mice, using astrocyte and macrophage/microglia markers, together with MSCs and EV-elective markers. BBB function was assessed using permeability tracers. After MSCs administration, neuroinflammation in EAE-affected mice appeared reduced and BBB features restored. MSCs were present inside the alveolar septa and were associated with EVs. We suggest that the trapped immunosuppressive MSCs release EVs into the circulation, which could immunomodulate encephalitogenic T cells, and/or cross the BBB to exert the anti-inflammatory effect observed in MSC-treated EAE-affected mice.

## A36 Wnt/β-catenin signaling is inhibited by sclerostin in the subfornical organ which results in a leaky barrier phenotype

### Finn Danker^1^, Fabienne Benz^1^,Elif Fidan^1^, Sonja Thom^1^, Ralf H. Adams^2^, M. Mark Taketo^3^, Stefan Günther^4^, Stefan Liebner^1^

#### ^1^Edinger Institute (Institute of Neurology), Goethe University Clinic, Frankfurt am Main, Germany;^2^Department of Tissue Morphogenesis, Max-Planck-Institute for Molecular Biomedicine, University of Münster, Germany;^3^Division of Experimental Therapeutics, Graduate School of Medicine, Kyoto University, Kyoto, Japan;^4^W. G. Kerckhoff Institute, Max Planck Institute for heart and lung research, Bad Nauheim, Germany

##### **Correspondence:** Stefan Liebner (stefan.liebner@kgu.de)

*Fluids and Barriers of the CNS* 2023, **20(Suppl 1)**: A36

The circumventricular organs (CVOs) are areas in the brain, lacking blood–brain barrier (BBB) properties and exhibit no Wnt/β-catenin signaling in endothelial cells (ECs). CVOs contribute to body homeostasis, such as water intake. Dominant, EC-specific β-catenin signaling changes vessels in the subfornical organ (SFO) to a tighter, BBB-like phenotype. Endothelial tightening results in increased neuronal activity in the SFO in water-restricted mice, suggesting a connection of barrier function with thirst and drinking behavior. Given the low or absent Wnt/β-catenin signaling in SFO ECs, active inhibition of the pathway can be suspected. We aimed (a) to identify and characterize the underlying mechanism for leaky vessel differentiation in the SFO and (b) to determine time- and physiology-dependent regulation of barrier function in the SFO. RNA sequencing and qPCR were conducted to identify potential targets of Wnt pathway inhibition. By fluorescence immunostaining (IHC) and fluorescent in situ hybridization (FISH), location of the gene expression of candidate genes was determined. Finally, microscopy images of IHC stainings were analyzed with bioinformatical tools in FIJI to quantify protein abundance of BBB biomarkers in the SFO in mice under various physiological circumstances. The Wnt inhibitor sclerostin (Scl) encoded by the Sost gene turned out to be specifically enriched in the SFO compared to microvessels of the cortex and the choroid plexus, which might have barrier-reducing effects on SFO vessels. Furthermore, claudin-5 protein levels increased and plasmalemma vesicle-associated protein (Plvap) levels decreased during aging, suggesting an age-dependent regulation of BBB properties. Water restriction, however, had the exact opposite effect.

## A37 A subcommissural organ-spondin derived peptide (NX210c) improves blood–brain barrier integrity in vitro

### Sighild Lemarchant^1^, Chris Greene^2^, Mélissa Sourioux^1^, Yann Godfrin^1,3^, and Matthew Campbell^2^

#### ^1^Axoltis Pharma, Lyon, France; ^2^Smurfit Institute of Genetics, Trinity College, The University of Dublin, Ireland; ^3^Godfrin Life-Sciences, Caluire-et-Cuire, France

##### **Correspondence:** Sighild Lemarchant (slemarchant@axoltis.com)

*Fluids and Barriers of the CNS* 2023, **20(Suppl 1)**: A37

Most of the CNS disorders or injuries imply blood–brain barrier (BBB) alterations that contribute to disease progression and functional impairments. The aim of this study was to evaluate the ability of a subcommissural organ-spondin-derived peptide (NX210c) to improve BBB integrity using an in vitro model. Mouse brain endothelial bEnd.3 cells were treated with NX210c (1, 10 and 100 µM) for up to 72 h. BBB integrity was assessed using transendothelial electrical resistance (TEER) and transwell permeability assay. The mRNA and protein expression levels of the main tight junction proteins (Claudin-5, Zonula Occludens-1 and Occludin) were measured by RT-qPCR, western-blot and immunocytochemistry. NX210c increased the TEER (+ 15%, + 31% for NX210c at 10, 100 µM for 72 h) and decreased the permeability of 40 kDa FITC-dextran across the cell monolayer (-53%, -50% for NX210c at 10, 100 µM for 72 h) in a dose-dependent manner. Although no modification of tight junction mRNA levels nor protein expression was observed, a 24-h treatment with NX210c (10, 100 µM) promoted the distribution of Claudin-5 at the tight junctions of endothelial cells. As a BBB integrity enhancer, NX210c may be an innovative drug candidate for the treatment of a large range of neurological diseases.

## A38 TRIM47 is crucial for the regulation of endothelial cell functions in brain

### Juliette Vaurs, Claire Peghaire, Valentin Delobel, Sébastien Rubin, Carole Proust, Béatrice Jaspard-Vinassa, Thierry Couffinhal, Cécile Duplàa

#### Biology of cardiovascular disease, University of Bordeaux, Inserm Franc

##### **Correspondence:** Juliette Vaurs (juliette.vaurs@inserm.fr)

*Fluids and Barriers of the CNS* 2023, **20(Suppl 1)**: A38

Cerebral small vessel disease (SVD) is a major contributor to vascular dementia (VD). Evidence indicates that blood brain barrier dysfunction may play a significant role in VD pathogenesis. Recently, we reported an inverse correlation between *TRIM47* expression in brain and extensive-SVD severity in a human genome wide association study. Our goal is to understand the role of TRIM47 in cerebral endothelial cells (EC). TRIM47 interacting effectors were searched by proximity labeling assay (BioID) in human brain EC. TRIM47 knockdown in EC allowed to explore TRIM47 role in functional assays, and to identify transcriptional response by RNA-sequencing. In vitro, TRIM47 knockdown decreases directed EC migration and delays EC adhesion process with loss of actin cortical reorganization and focal adhesion contacts. Put together, RNA sequencing and BioID results indicate that TRIM47 knockdown in brain EC, represses the expression of genes associated with cytoskeleton and NRF2 antioxidant pathway through a potential interaction with KEAP1, an important player in focal adhesion and cytoskeleton organization. These results suggest that endothelial TRIM47 is a key regulator of actin cytoskeleton organization through KEAP1/NRF2 signaling pathway and might be protective from oxidative stress in brain EC.

## A39 Identification of new interaction partners of the tight junction protein occludin

### Victoria Kaupp^1^, Lucie Y Li^2^, Markus Höltje^2^, Patrick Meybohm^1^, Malgorzata Burek^1^

#### ^1^University of Würzburg, University Hospital Würzburg, Department of Anaesthesiology, Intensive Care, Emergency and Pain Medicine, Würzburg, Germany; ^2^Institute of Integrative Neuroanatomy Berlin, Charité-Universitätsmedizin Berlin, corporate member of Freie Universität Berlin, Humboldt-Universität zu Berlin, and Berlin Institute of Health, Berlin, Germany

##### **Correspondence:** Victoria Kaupp (Kaupp_V@ukw.de)

*Fluids and Barriers of the CNS* 2023, **20(Suppl 1)**: A39

Occludin is found in almost every barrier-forming tissue, including the blood–brain barrier (BBB). As part of the tight junction-associated MARVEL protein (TAMP) family, occludin seems to play a complex regulatory role in tight junction physiology, yet the mechanisms of its contribution to BBB tightness have not yet been sufficiently characterized. To elucidate the role of occludin in BBB physiology, we examined occludin interaction partners using different methods: first, a yeast two-hybrid screen led us to various possible protein–protein interactions, which were then confirmed by immunoprecipitation. The expression of identified interaction partners was characterized in primary brain microvascular endothelial cells, various in vitro BBB models and isolated brain capillaries. Co-staining of occludin with its interaction partners was performed on brain slices. In addition, immunofluorescence staining was used to visualize the interaction partners in different cell lines. The immunoprecipitation itself was performed in three steps: (1) analysis of HEK293 cells overexpressing the proteins of interest by double transfection, (2) analysis of CaCo2 cells with endogenous occludin overexpressing the interaction partner by single transfection, (3) immunoprecipitation of endogenous proteins in CD34 + hematopoietic stem cells-derived human endothelial cells and hCMEC/D3 cells used as BBB models. Next, we constructed knockout cell lines by deleting interaction partners alone or in combination with occludin using CRISPR/Cas9 method. The effects of knockout on BBB properties are currently being tested. Our results show that occludin is involved in numerous signaling pathways and directly interacts with many proteins. The detailed role of these interactions needs to be evaluated in future studies.

## A40 Junctional adhesion molecules: a play critical role in the thromboembolic occlusion and stroke injury in Alzheimer disease

### Svetlana M. Stamatovic^1^, Richard F. Keep^2,3^, and Anuska V. Andjelkovic^1,2^

#### ^1^Department Pathology, ^2^Neurosurgery and ^3^Molecular and Integrative Physiology, University of Michigan, Ann Arbor, MI, USA

##### **Correspondence:** Svetlana M Stamatovic (sstamato@med.umich.edu)

*Fluids and Barriers of the CNS* 2023, **20(Suppl 1)**: A40

Accumulating evidence pinpoints the frequent co-occurrence of stroke and Alzheimer’s disease (AD) pathology. Our recent transcriptomic and proteomic profiling of microvessels with amyloid deposition revealed high expression of junctional adhesion molecule (JAM)-A, a tight junction protein, which also act as adhesion molecule involved in brain endothelial cell (BEC)-platelet and brain endothelial cell-leukocyte interactions in pathological conditions. The study is aiming to elucidate whether JAM-A plays a role in platelet- brain endothelial cell interactions in thromboemboli lodging in the setting of AD. The effect of JAM-A on the stroke injury was assessed in a thromboembolic (TE) stroke model induced in murine AD model with amyloid vasculopathy in three conditions modifying JAM-A expression: a) JAM-A absence in BECs (3xTg mice x JAM-A^−/−^Cldn5^Cre^), b) absence of JAM-A on the platelets in thromboemboli (CD41 + /JAM-A-), and c) absence of JAM-A from BECs and platelets (3xTgxJAM-A KO). The infarct size was assessed by MRI T2 imaging, neurological deficit by batteries of neurobehavioral tests and TE emboli lodging by immunohistology. Absence of JAM-A either in BECs and/or platelets, improved stroke outcome (infarct size, neurological deficit, inflammatory response). JAM-A absence from the BECs significantly reduce the leukocyte infiltration after stroke. Modifying the JAM-A expression in platelets predominately affected the lodging of thromboemboli, reducing the number of the occluded vessels by 40%. The JAM-A/JAM-A interaction between brain endothelial cells and CD41 + platelets in emboli facilitate their lodging in amyloid-effected blood vessels and JAM-A on leukocytes interactions driving the inflammatory response of brain endothelial cells and perivascular cells.

## A41 Truncated LRP1 receptor-based transport system at the blood–brain barrier

### Magdalena Kurtyka, Laura Fritzen, Claus U. Pietrzik

#### Institut for Pathobiochemistry, University Medical Center Mainz, Mainz, Germany

##### **Correspondence:** Magdalena Kurtyka (mkurtyka@uni-mainz.de)

*Fluids and Barriers of the CNS* 2023, **20(Suppl 1)**: A41

Transport of molecules across the blood–brain barrier (BBB) is strictly regulated by both carrier and receptor-based transport systems. The number of known BBB-enriched receptors allowing delivery of therapeutics into the brain or removal or toxic molecules, such as amyloid beta, is scarce. Here we propose an artificial low density lipoprotein receptor-related protein 1 (LRP1) mini-receptor delivered via murine brain endothelium-specific adeno-associated virus (AAV-BR1) [1] as a target for neuroactive drug distribution in the central nervous system. The purpose of this study is to establish a therapeutic-oriented tool for transport of molecules across the BBB. HA/myc-tagged LRP1 mini constructs consisting of truncated alpha-chain and full beta-chain of human LRP1 were cloned into AAV-BR1 plasmid for recombinant AAVs (rAAVs) production. Human kidney embryonic cells (HEK293T) were transduced with rAAVs (MOI 100,000) and lysed on a day 4. post-transduction. 2-months old mice intravenously injected with rAAVs (1E11 viral particles per mouse) were sacrificed 4 weeks post-injection. SDS-PAGE and Western blot was performed to analyze expression of LRP1mini in HEK293T cells, isolated brain capillaries and brain-depleted brains. We show that LRP1 mini receptor is expressed both in vitro in transduced HEK293T cells as well as in vivo in injected adult mice. Our results demonstrate that LRP1mini can be successfully delivered at the BBB of adult mice. More studies are needed to evaluate the functionality of the receptor as a BBB transporter.

## Reference


Körbelin J, Dogbevia G, Michelfelder S, Ridder DA, Hunger A, Wenzel J et al. A brain microvasculature endothelial cell-specific viral vector with the potential to treat neurovascular and neurological diseases. EMBO Mol Med. 2016;8(6):609–25. https://doi.org/10.15252/emmm.201506078.

## A42 Changes in the glucose and monocarboxylate transporters in hippocampi of female rats subjected to the western diet and forced physical activity

### Konstancja Grabowska^1^, Daniela Liskiewicz^1^, Patrick Meybohm^2^, Małgorzata Burek^2^, Andrzej Małecki^1^, and Marta Nowacka-Chmielewska^1^

#### ^1^Laboratory of Molecular Biology, Institute of Physiotherapy and Health Sciences, Academy of Physical Education, Katowice, Poland; ^2^University Hospital Würzburg, Department of Anaesthesiology, Intensive Care, Emergency and Pain Medicine, Würzburg, Germany

##### **Correspondence:** Marta Nowacka-Chmielewska (m.nowacka@awf.katowice.pl)

*Fluids and Barriers of the CNS* 2023, **20(Suppl 1)**: A42

Western diet (WD)-induced adverse effects in the brain seem to be related to disturbances of brain energy metabolism. The disturbances of glucose homeostasis may result from the well-recognized development of insulin resistance, but may also be caused by impaired glucose transport into the brain. It has been shown that GLUTs haploinsufficiency leads to the age-related impairment of cerebral blood flow, increasing the permeability of the blood–brain barrier (BBB) and cognitive impairment. Studies published thus far show that the energy challenge caused by exercise can improve cellular bioenergetics and attenuate inflammation processes. However, little is known about why it has such a profound effect on the brain. The aim of the study was to assess the impact of simultaneous exposure to a WD and wheel-running on the hippocampal level of tight junctions and proteins related to glucose transport across the BBB. 9-weeks old female Long Evans rats alongside standard rodent chow received snacks typical for human WD for 6 weeks. During this time seven animals were also subjected to forced physical activity (wheels with electric motor; 5 days a week, 1 h daily). Animals in the control group received standard rodent chow and did not have access to running wheels. At the end of the experiment, hippocampi were isolated, weighed and stored at − 80 °C until western blotting analysis. The WD significantly decreased ZO-1 (p = 0.019), but no changes were observed in levels of occludin. The level of GLUT8, which is expressed mainly in neurons, was increased in the hippocampi of exercised animals fed with a WD (p < 0.0001). In the exercised rats fed with a WD, a 0.87-fold decrease in the level of hippocampal MCT4 (p = 0.009) was observed, while the level of MCT5 was increased as compared to the not exercised WD rats (p = 0.0034).

Our results suggest that physical activity may increase the availability of glucose for neurons by increasing glucose transport into the cells through MCT5, which supports earlier evidence that energy challenges stimulate pathways associated with the usage of alternative energy sources such as monocarboxylates.

**Grant Support:** This research is supported by National Science Centre Grant no. 2015/19/D/NZ7/02408.

## A43 Interaction between small extracellular vesicles and human blood–brain barrier in vitro models

### Ana Špilak^*^, Adrián Klepe^*^, Sophia Theresa Kriwanek, Andreas Brachner, Christa Nöhammer, Winfried Neuhaus

#### Center for Health and Bioresources, Competence Unit Molecular Diagnostics, AIT Austrian Institute of Technology GmbH, Vienna, Austria

##### **Correspondence:** Winfried Neuhaus (winfried.neuhaus@ait.ac.at)

*Equally contributed

*Fluids and Barriers of the CNS* 2023, **20(Suppl 1)**: A43

Small extracellular vesicles (sEVs) are released by many cells, among them cancer cells. Heterogeneous sEVs actively interact with biological barriers such as the blood–brain barrier (BBB) [1] and play a pivotal role in oncogenesis, angiogenesis, and cancer metastasis [2]. The communication between human brain capillary endothelial cells (BCECs) and cancer-derived sEVs is not understood yet. Therefore, we aim to investigate this communication. Small EVs derived from cell lines (HEK293, DU145, etc.) were indirectly (transfection, GFP-GPI anchor) or directly (membrane permeable dyes) fluorescently labelled and applied either to transwell models (transport), or on coverslips (uptake) using hCMEC/D3 immortalised cell line [3] and human induced pluripotent stem cell-derived brain capillary endothelial-like cells, hiPSC-BCECs [4]. To follow the distribution and localisation of stained sEVs, flow cytometry and fluorescence microscopy were used. 1–10% of GFP + sEVs from transfected cells permeated through blanks and differentiated hiPSC BBB models. Fluorescence microscopic images indicated either membrane surface binding or internalisation of CellTracker Orange (CTO) labelled sEVs in hCMEC/D3 cells. Flow cytometric analyses confirmed the interaction between CTO labelled sEVs and both human BBB models. Additionally, applied proinflammatory cytokines led to barrier breakdown, and resulting changes of uptake of sEVs in BBB models were investigated. Our results confirm the interaction between cancer-derived sEVs and human BBB models, albeit there is need for further investigations regarding transport pathways using advanced high-resolution microscopy to elucidate the cellular mechanisms of sEV-cell communication.

**Grant Support:** This work was founded by the European Union’s Horizon 2020 research and innovation programme (Marie Skłodowska-Curie project No 860303). We further gratefully acknowledge the financial support provided by the Austrian Science Fund FWF (project P 34137-B).

## References


Kuroda H, Tachikawa M, Yagi Y, Umetsu M, Nurdin A, Miyauchi E et al. Cluster of Differentiation 46 Is the Major Receptor in Human Blood–Brain Barrier Endothelial Cells for Uptake of Exosomes Derived from Brain-Metastatic Melanoma Cells (SK-Mel-28). Mol Pharm. 2019;16(1):292–304. https://doi.org/10.1021/acs.molpharmaceut.8b00985.Špilak A, Brachner A, Kegler U, Neuhaus W, Noehammer C. Implications and pitfalls for cancer diagnostics exploiting extracellular vesicles. Adv Drug Deliv Rev. 2021;175:113819. https://doi.org/10.1016/j.addr.2021.05.029.Gerhartl A, Pracser N, Vladetic A, Hendrikx S, Friedl HP, Neuhaus W. The pivotal role of micro-environmental cells in a human blood–brain barrier in vitro model of cerebral ischemia: functional and transcriptomic analysis. Fluids Barriers CNS. 2020;17(1):19. https://doi.org/10.1186/s12987-020-00179-3.Appelt-Menzel A, Cubukova A, Günther K, Edenhofer F, Piontek J, Krause G et al. Establishment of a Human Blood–Brain Barrier Co-culture Model Mimicking the Neurovascular Unit Using Induced Pluri- and Multipotent Stem Cells. Stem Cell Reports. 2017;8(4):894–906. https://doi.org/10.1016/j.stemcr.2017.02.021. Epub 2017 Mar 23. PMID: 28344002; PMCID: PMC5390136.

## A44 Blood–brain barrier: peptide shuttles for drug delivery into the CNS

### Paula Schwarz^1^, Kirtikumar B. Jadhav^2^, Christian W Gruber^1^, and Roland Hellinger^1^

#### ^1^Center for Physiology and Pharmacology, Medical University of Vienna, Vienna, Austria; ^2^Institute of Biological Chemistry, University of Vienna, Vienna

##### **Correspondence:** Roland Hellinger (roland.hellinger@meduniwien.ac.at)

*Fluids and Barriers of the CNS* 2023, **20(Suppl 1)**: A44

The blood–brain-barrier (BBB) is one of the main obstacles in the development of new therapeutics for diseases located in the central nervous system as most large molecules and over 98% of small molecules do not cross the BBB [1]. Exploiting endogenous receptor mediated transcytosis by using natural peptide ligands or derivatives thereof as “Trojan Horses” to transport active substances across the BBB has been proven a promising strategy to overcome limited transport. For example, the peptide shuttle Angiopep-2 has already entered clinical trials [2]. However, peptidic therapeutics are prone to proteolytic inactivation and a resulting short serum half-life restricts their application mostly to intravenous injection. As a solution for the proteolytic instability of linear peptides, bioactive peptide sequences can be incorporated into natural stabilized peptide scaffolds, e.g., cyclic cystine-rich knot (CCK) peptides, with the anticipated result that these hybrid molecules conserve bioactivity and stability [3]. In this study, we designed novel BBB shuttles by grafting a series of BBB active peptide sequences into a stabilizing cyclic scaffold. These hybrid molecules were tested for BBB transport for which a BBB transwell screening assay using the human cerebral endothelial cell line hCMEC/D3 was established. The probes were chemically and functionally characterized and compared to prototypical BBB permeable peptides. Our results indicate that peptide scaffolds are useful to incorporate BBB active epitopes while maintaining receptor mediated transcytosis for transport into the CNS. Our finding may open avenues for the design of new multi-functional BBB peptide shuttles with extended serum half-life.

## References


Kumthekar P, Tang SC, Brenner AJ, Kesari S, Piccioni DE, Anders C et al. ANG1005, a Brain-Penetrating Peptide-Drug Conjugate, Shows Activity in Patients with Breast Cancer with Leptomeningeal Carcinomatosis and Recurrent Brain Metastases. Clin Cancer Res. 2020;26(12):2789–2799. https://doi.org/10.1158/1078-0432.CCR-19-3258.Pardridge WM. The blood–brain barrier: bottleneck in brain drug development. NeuroRx. 2005;2(1):3–14. https://doi.org/10.1602/neurorx.2.1.3.Muratspahić E, Tomašević N, Koehbach J, Duerrauer L, Hadžić S, Castro J et al. Design of a Stable Cyclic Peptide Analgesic Derived from Sunflower Seeds that Targets the κ-Opioid Receptor for the Treatment of Chronic Abdominal Pain. J Med Chem. 2021;64(13):9042–9055. https://doi.org/10.1021/acs.jmedchem.1c00158.

## A45 Does the proton-coupled organic cation antiporter contribute to brain capillary endothelial cell uptake of triptans?

### Nana Svane^1^, Alberte Pedersen^1^, Burak Ozgür^1^, Lasse Saaby^1,2^, Mie Kristensen^1^, Peer Tfelt-Hansen^3^, Birger Brodin^1^

#### ^1^Department of Pharmacy, University of Copenhagen, Denmark; ^2^Bioneer: FARMA, Bioneer A/S, Copenhagen, Denmark; ^3^Danish Headache Center, Department of Neurology, Rigshospitalet-Glostrup, University of Copenhagen, Glostrup, Denmark

##### **Correspondence:** Nana Svane (nana.svane@sund.ku.dk)

*Fluids and Barriers of the CNS* 2023, **20(Suppl 1)**: A45

Triptans are 5-HT_1_ receptor agonists, used to treat migraine attacks. Most triptans are regarded to be unable to cross the blood–brain barrier (BBB). However, some studies indicate central effects of this class of drug compounds. Triptans are generally hydrophilic, with a positive net charge, and are thus not expected to cross the BBB passively by transcellular diffusion. A proton-coupled organic cation (H^+^/OC) antiporter has been shown to facilitate brain uptake of multiple drug compounds. The aim of the present project was to investigate if the putative H^+^/OC antiporter may mediate transport of triptans across the BBB. Uptake studies were performed in a human in vitro BBB model (hCMEC/D3) using radiolabeled [^3^H]-pyrilamine, non-labeled oxycodone and non-labeled triptans. Oxycodone, almotriptan, rizatriptan and zolmitriptan inhibited the cellular uptake of [^3^H]-pyrilamine with IC_50_ values of 201 ± 36, 278 ± 105, 719 ± 198 and 1731 ± 990 µM, respectively. [^3^H]-pyrilamine uptake remained unaffected in the presence of naratriptan, sumatriptan and frovatriptan. Almotriptan uptake studies, with LC–MS detection, showed that almotriptan exhibited time-, concentration- and pH-dependent uptake into hCMEC/D3 cells. In addition, almotriptan exhibited a lower cellular uptake in the presence of pyrilamine. Almotriptan, rizatriptan and zolmitriptan interacts with the H^+^/OC antiporter. Uptake of almotriptan was demonstrated using LC–MS. This suggest that the H^+^/OC antiporter may be involved in the blood-to-brain transport of certain triptans.

## A46 Optimization of a CNS target-activated TNFR2 agonist delivery through transferrin receptor-mediated transport across brain barriers for neurodegenerative diseases treatment

### Sabrina Petralla^1,6^, Valentina Pegoretti^2,6^, Gavin Fullstone^2,3,6^, Fatemeh Dabbagh^4,6^, Philipp Kuhn^5,6^, Thomas Schirrmann^5,6^, André Frenzel^5,6^, Jonas Kügler^5,6^, Christian Schwerk^4,6^, Horst Schroten^4,6^, Roman Fischer^2,3,6^, Roland Kontermann^2,3,6^, Markus Rehm^2,3,6^, Gert Fricker^1,6^

#### ^1^Institute of Pharmacy and Molecular Biotechnology, University of Heidelberg, Heidelberg, Germany; ^2^Institute for Cell Biology and Immunology, University of Stuttgart, Stuttgart, Germany; ^3^Stuttgart Research Centre Systems Biology (SRCSB), University of Stuttgart, Stuttgart, Germany; ^4^Pediatric Infectious Diseases, Department of Pediatrics, Medical Faculty Mannheim, Mannheim, Germany; ^5^YUMAB GmbH, Braunschweig, Germany; ^6^BRAINAIM Consortium, BMBF Gezielter Wirkstofftransport

##### **Correspondence:** Sabrina Petralla (s.petralla@uni-heidelberg.de)

*Fluids and Barriers of the CNS* 2023, **20(Suppl 1)**: A46.

Effective drug delivery into the central nervous system (CNS) is one of the major challenges in the treatment of neurodegenerative diseases due to selectivity of the brain barriers (the blood brain barrier [BBB] and the blood-cerebrospinal fluid [CSF] barrier [BCSFB]) that prevent most small drugs and macromolecules from reaching the CNS [1]. To overcome these highly selective barriers, receptor-mediated transcytosis (RMT) seems to be the most promising pathway to deliver biologics into the CNS. A progenitor molecule of ARTOS, a potent agonist of the tumor necrosis factor receptor 2 (TNFR2), has demonstrated great potential in pre-clinical studies in promoting neuroprotection and immunomodulation [2, 3], but lacks efficient transport into the CNS. In order to develop an optimized CNS-targeted variant of ARTOS by inducing RMT via the transferrin receptor (TfR) as a promising therapeutic for neurodegenerative diseases, a multidisciplinary approach was designed, combining expertise in drug delivery, biologic drug development and in silico strategies. The approach aims to minimize unwanted peripheral TNFR2 activation, to improve CNS delivery, and to unfold neuroprotective ARTOS bioactivity on-target upon delivery. Taken together, the data will have a significant impact to counteract unmet needs in neurodegenerative diseases, including Alzheimer’s and Parkinson’s disease which have an increasing relevance in an aging society, whilst simultaneously providing a strategy for future optimization of several CNS-therapeutics.

**Grant Support:** This work was supported by funding from the German Federal Ministry of Education and Research, BMBF (FKZ: 16GW0325 (BrainAim)).

## References


William M Pardridge. Blood–Brain Barrier and Delivery of Protein and Gene Therapeutics to Brain. Front Aging Neurosci. 2020;11:373. https://doi.org/10.3389/fnagi.2019.00373.Yun Dong, Roman Fischer, Petrus J W Naudé, Olaf Maier, Csaba Nyakas, Maëlle Duffey et al. Essential protective role of tumor necrosis factor receptor 2 in neurodegeneration. Proc Natl Acad Sci U S A. 2016;113(43):12304–12309. https://doi.org/10.1073/pnas.1605195113.Roman Fischer, Tanja Padutsch, Valerie Bracchi-Ricard, Kayla L Murphy, George F Martinez, Niky Delguercio et al. Exogenous activation of tumor necrosis factor receptor 2 promotes recovery from sensory and motor disease in a model of multiple sclerosis. Brain Behav Immun. 2019;81:247–259. https://doi.org/10.1016/j.bbi.2019.06.021.

## A47 Pericyte hypoxia-inducible factor-1 is required for blood–brain barrier recovery during the long-term stroke

### Chih-Chieh Tsao^1,2^, Diana Kindler^3^, Christina Köster-Hegmann^2^, Daniel Razansky^3^, and Omolara Ogunshola^1,2^

#### ^1^Zurich Center of Integrative Human Physiology, University of Zurich, Zurich, Switzerland; ^2^Institute for Veterinary Physiology, University of Zurich, Zurich, Switzerland; ^3^Institute for Biomedical Engineering, ETH Zurich, Zurich, Switzerland

##### **Correspondence:** Chih-Chieh Tsao (chih-chieh.tsao@uzh.ch)

*Fluids and Barriers of the CNS* 2023, **20(Suppl 1)**: A47

Dysfunction of the blood–brain barrier (BBB), a multicellular structure comprised of vascular endothelium, pericytes (PC) and astrocyte end-feet, leads to worse stroke outcome. Targeting BBB has been accepted by increasing number of researchers and is expected to be a novel strategy of stroke treatment. As PC significantly contribute to ischemic BBB stability, targeting these perivascular cells could provide a new paradigm for modulating barrier integrity and improving stroke outcome. Hypoxia inducible factor-1 (HIF-1) regulates cell stress adaptive responses under hypoxia/ischemia. Our previous study showed loss of pericytic HIF-1 function improves ischemic barrier stability and acute stroke outcome. We herein ask if the early positive effects of HIF-1 loss of function (LoF) will last in extended recovery stroke phases. We subjected an inducible PC-targeted HIF-1 LoF mouse line (SMMHC-Cre^ERT2^; HIF-1⍺^flox/flox^) to transient middle cerebral artery occlusion (tMCAo) and monitored brain damage, BBB permeability and neurobehavioral function during the longer reperfusion period. Surprisingly, at later stroke stages, we discovered a complete reversal of early beneficial effects of HIF-1 LoF. Stroke HIF-1 LoF mice had enhanced brain shrinkage and fewer surviving neurons in correlation with more behavioral deficits. A significantly worse barrier stability was also observed in HIF-1 LoF groups compared to WT controls. Thus, our data suggests PC-mediated HIF-1 signaling is essential for the barrier remodeling at late stroke phases. Underlying mechanisms are currently being explored.

